# FTO‐mediated m^6^A modification of *SOCS1* mRNA promotes the progression of diabetic kidney disease

**DOI:** 10.1002/ctm2.942

**Published:** 2022-06-22

**Authors:** Qiang Sun, Houfa Geng, Meng Zhao, Yang Li, Xi Chen, Qian Sha, Peng Lai, Daoquan Tang, Dongzhi Yang, Jun Liang, Mengzhe Guo

**Affiliations:** ^1^ Jiangsu Key Laboratory of New Drug Research and Clinical Pharmacy Xuzhou Medical University Xuzhou Jiangsu China; ^2^ Department of Endocrinology Xuzhou Central Hospital, Xuzhou Clinical School of Nanjing Medical University Xuzhou Jiangsu China; ^3^ Institute of Thoracic Oncology and Department of Thoracic Surgery West China Hospital, Sichuan University Chengdu Sichuan China

**Keywords:** diabetic kidney disease, m^6^A modification, single‐cell omics

Dear Editor,

N^6^‐methyladenosine (m^6^A) is the most prominent and frequent internal messenger RNA (mRNA) modification and plays diverse roles in regulating functions of modified transcripts.^1^ However, the role of m^6^A modification in kidney disease remains rarely understood, especially at the onset of diabetic kidney disease (DKD).^2^ Here, we delineate the biological role of FTO‐mediated m^6^A modification in DKD through imaging mass cytometry (IMC), LC/MS, and RNASeq methods. The results show that the loss of m^6^A levels by overexpressing FTO recapitulated human DKD by increasing the expression of suppressors of cytokine signalling 1 (SOCS1) protein level to alleviate inflammation response and kidney injury. Thus, FTO maybe a potential therapeutic target for DKD patients.

To quantify the m^6^A level at cellular and spatial levels, we designed an IMC panel specific to kidney histology and used this to analyse kidney biopsies (Figure 1A and Table S1). IMC integrates IHC using metal isotope‐tagged antibodies with laser ablation and mass‐spectrometry‐based detection to produce high‐dimensional images,^3^ which allow simultaneously quantified the m^6^A levels and its regulators. We identified 17 676 cells and quantified the levels of m^6^A, regulators, and spatial characteristics at single‐cell level (Figures 1 and S1). We identified five dominant cell clusters of proximal tubules, distal convoluted tubule, glomerulus (Glom) endothelial, macrophage, and stromal cell populations.^4^ IMC and IHC data demonstrated that the m^6^A levels were significantly increased in several types of cells, and FTO expression was significantly reduced (Figure 2). The results suggest that FTO plays important roles in m^6^A dynamics in DKD.

**FIGURE 1:**
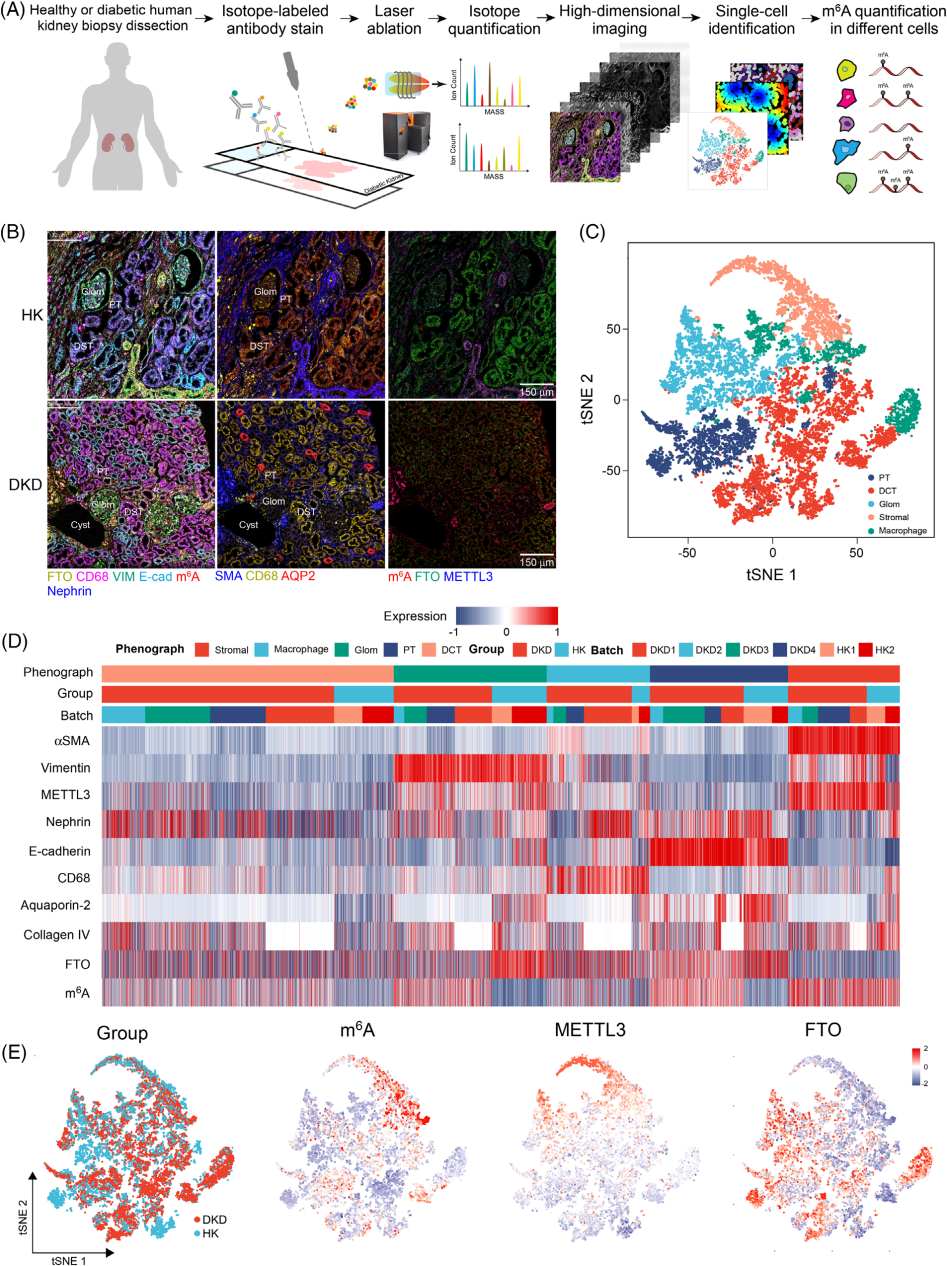
Single‐cell phenotypes in high‐dimensional histopathology of diabetic kidney: (A) schematic of imaging mass cytometry (IMC) acquisition of multiplexed images from four patients with diabetic kidney disease (DKD) and the analyses of single‐cell phenotypes, meta‐clusters, and architecture; (B) summary example images from different patients of the analysed cohort; (C) map using *t*‐distributed stochastic neighbour embedding (*t*‐SNE) of 17 676 subsampled single cells from high‐dimensional images of kidneys coloured by cell‐type meta‐cluster identifier; (D) heat map showing the *z*‐scored marker expression of each cell; (E) *t*‐SNE plot showing the group of DKD or healthy kidney (HK) and the expression of m^6^A, METTL3, and FTO in each cell

**FIGURE 2:**
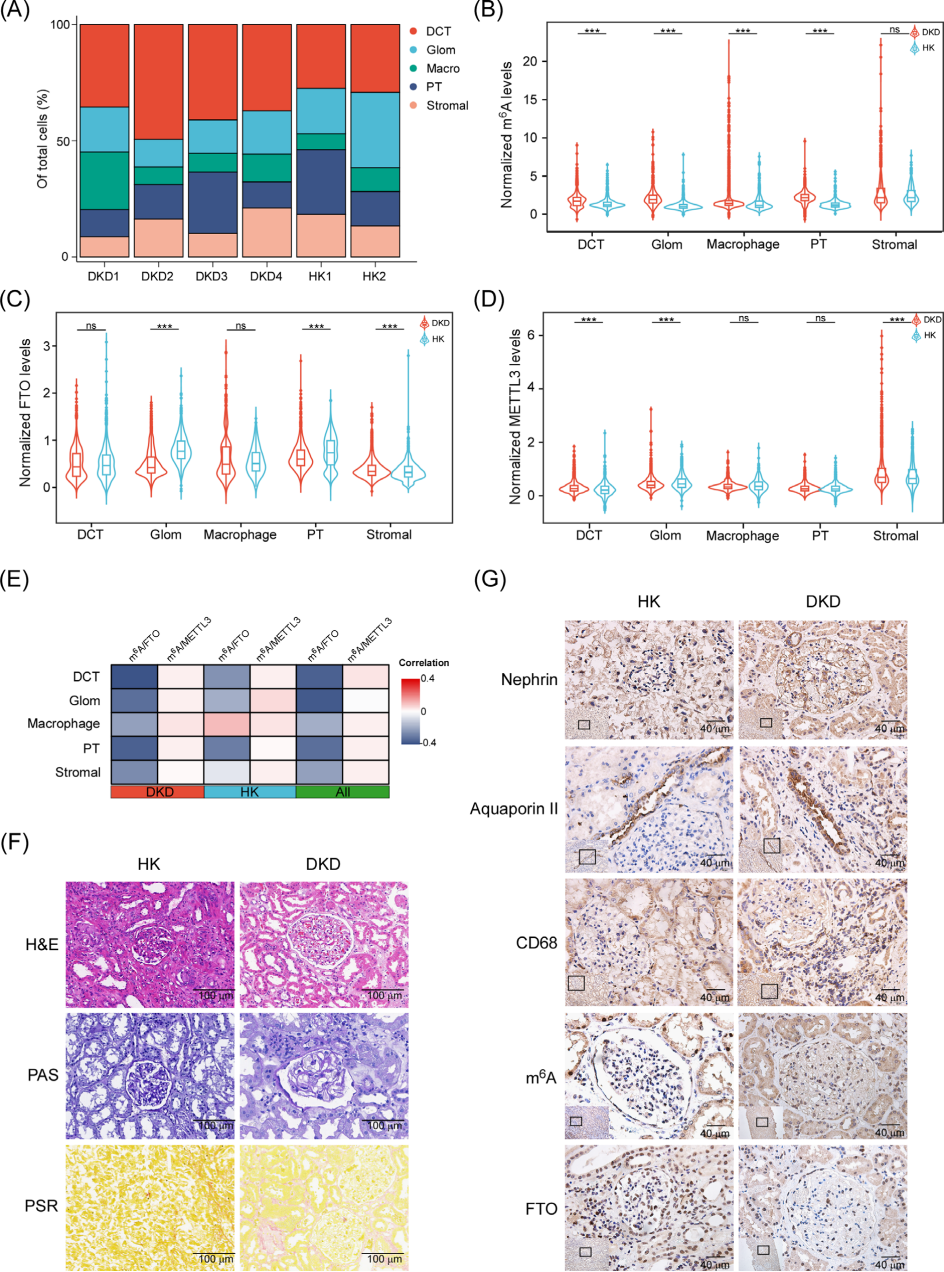
Imaging mass cytometry (IMC) analysis identifies increased m^6^A modification levels in various cells in diabetic kidney disease (DKD) patients: (A) the composition of different cells in different samples; (B–D) violin plot showing the expression of m^6^A (B), METTL3 (C), and FTO (D) in different types of cells across DKD and healthy kidney (HK) samples; (E) heat map showing correlation between m^6^A and METTL3/FTO in different types of cells across DKD and HK samples; (F) representative images of H&E, PAS, and PSR analyses of kidney biopsies from DKD or health kidneys; (G) representative images of IHC analyses of indicated proteins of kidneys from DKD or health kidneys. Statistical analyses were performed by two‐tailed Mann–Whitney *U* test. ns, not significant; ***p < .001

Further analysis revealed that serum m^6^A levels were significantly upregulated in T2D and DKD patients (Figures S2 and S3a). RNASeq data showed that FTO was significantly downregulated in T2D and DKD patients, whereas other regulators remained unchanged (Figures S3b and S4). Further data also showed that the m^6^A levels remained significantly upregulated in T2D and DKD patients, and FTO level was negatively correlated with m^6^A levels (Figure S3c–e). Furthermore, re‐analyses of public datasets revealed that FTO was significantly decreased in DKD or uremina patients (Figure S3f–h). To further investigate the role of FTO in DKD pathogenesis, high‐concentration glucose (HG) treatment was used to simulate the phenotypes in DKD.^5^ HG treatment significantly reduced FTO expression, whereas the m^6^A RNA level was significantly increased (Figure S5a–d). Moreover, other regulators remained unchanged after the HG treatment (Figure S6). FTO overexpression or knock‐down significantly reduced or augmented the m^6^A levels, respectively (Figure S5e–h). HG often triggers glucose‐response transcriptional factor ChREBP expression,^6^ further analysis revealed that the FTO promoter had several ChREBP‐binding sites, indicating HG may suppress FTO expression via activating ChREBP (Figure S5i). Together, these data reveal that the m^6^A modification levels are increased due to FTO downregulation in DKD.

To investigate the molecular mechanism by how dysregulated FTO is involved in DKD. We performed MeRIP‐seq in HMC after FTO overexpression, and the results showed that m^6^A peaks were significantly enriched at the 3′‐UTR region (Figure 3A,C) and were characterized by the RAACH motif (Figure 3B). Overall, 25 genes were affected at both RNA expression and m^6^A levels. Pathway enrichment analyses revealed that the genes affected are involved in inflammation (Figure 3D,E and Tables S6 and S7). More specifically, the m^6^A level of SOCS1, a key regulator of inflammation, was significantly reduced after FTO overexpression (Figure 3F), which was confirmed by re‐analyses of public datasets (Figure S7a). MeRIP‐qPCR results showed that *SOCS1* m^6^A level was significantly reduced on FTO overexpression (Figure 3G). Further data revealed that SOCS1 protein level was significantly increased when FTO was overexpressed (Figure 3H). *SOCS1* mRNA expression was also positively associated with FTO expression (Figure S7b). Moreover, SOCS1 expression was significantly suppressed after FTO knock‐down (Figure S7c). The inhibition of FTO induced by HG treatment could also result in the reduced expression of SOCS1 (Figure S7d). Further results showed that demethylation‐inactive mutant FTO (H231A and D233A) had no effects on both m^6^A and protein level of SOCS1 compared with wild‐type FTO (Figures S7e and 3I).Moreover, FTO‐RIP‐qPCR assays showed the direct binding of FTO on *SOCS1* mRNA (Figure 3J). Our findings indicate that FTO can increase the expression of SOCS1 via removing its m^6^A.

**FIGURE 3:**
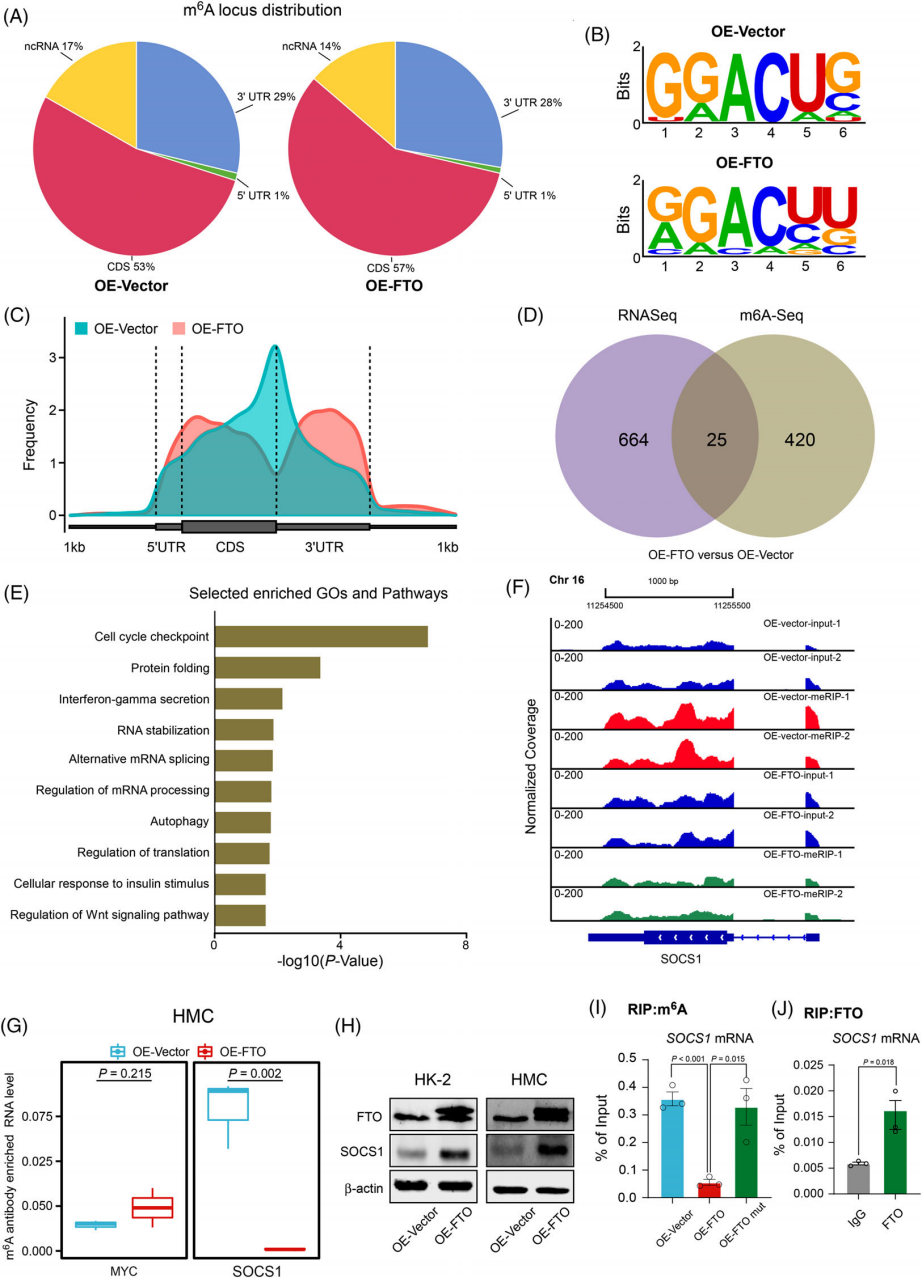
FTO‐mediated m^6^A modification of suppressors of cytokine signalling 1 (*SOCS1*) messenger RNA (mRNA) decreases its protein expression due to loss of FTO: (A) pie chart of m^6^A locus distribution in controlsand FTO overexpression HMC cells; (B) m^6^A enriched for known consensus motif RAACH using HOMER; (C) m^6^A‐enriched peak distribution on the metagene using guitar; (D) RNA‐seq and MeRIP‐seq identified differentially expressed genes or m^6^A peaks in FTO stable overexpression HMC cells when compared with their corresponding controls; (E) enriched pathway analyses of significantly altered m^6^A peaks after FTO overexpression. *p* Values were calculated according to the hypergeometric test based on the number of physical entities present in both the predefined set and user‐specified list of physical entities; (F) the m^6^A abundances on *SOCS1* mRNA transcripts in HMC cells as detected by MeRIP‐seq were plotted; (G) MeRIP‐qPCR analysis was employed to demonstrate FTO‐mediated SOCS1 m^6^A modifications. The m^6^A modification of SOCS1 was decreased on the upregulation of FTO; (H) Western blot analyses of indicated proteins in HMC or HK‐2 cells after FTO overexpression; (I) meRIP‐PCR analysis of *SOCS1* mRNA level in HMC cells after overexpressing wild‐type or mutant FTO wild‐type; (J) FTO‐RIP‐PCR analysis of *SOCS1* mRNA level in HMC cells. Data are represented as mean  ± s.e.m. Statistical analyses were performed by two‐tailed unpaired student *t*‐tests and corrected for multiple comparisons using the Holm‐Sidak method.

Inflammation plays vital roles in DKD, and SOCS1 is considered an important inflammation regulator.^8^, ^9^ Network and GSEA analyses showed that inflammation‐related pathways including the JAK‐STAT pathway were significantly repressed after FTO overexpression (Figure S8a,b). Moreover, FTO overexpression significantly inhibited inflammation via inhibiting JAK2/STAT3 phosphorylation. In contrast, FTO knock‐down and HG aggravated DKD by promoting JAK2/STAT3 phosphorylation (Figure S8c–e). The rescue assay showed that p‐JAK2 and p‐STAT3 levels were significantly inhibited after SOCS1 knock‐down (Figure S8f). Collectively, these results suggest that the JAK‐STAT signalling pathway is activated due to the decreased FTO expression.

We generated the *db*/*db* mice that were most commonly used T2D/DKD model.^10^ Intriguingly, injecting fto‐overexpressing lentivirus significantly alleviated kidney damage, indicating that fto might be a potential therapeutic target (Figure S9a–g). LC/MS results showed that m^6^A levels were higher in *db*/*db* mice, and m^6^A levels were decreased after injecting FTO‐overexpressing lentivirus (Figures 4A and S9h). qRT‐PCR and analyses of public microarray data showed that fto expression was significantly reduced in various mouse models (Figure S9i–m). WB and IHC results showed that both fto and socs1 were significantly reduced in *db*/*db* mice, which led to inflammation response via promoting jak2 and stat3 phosphorylation (Figures 4B,C and S10). Surprisingly, fto overexpression significantly increased socs1 expression which alleviated inflammation response as the IHC and WB data showed (Figures 4B,C and S10). The H&E, PAS, SR, and IHC data further indicated that fto overexpression attenuated kidney injuries and fibrosis. Thus, our data suggest that fto overexpression can alleviate kidney injury via suppression of inflammation.

**FIGURE 4:**
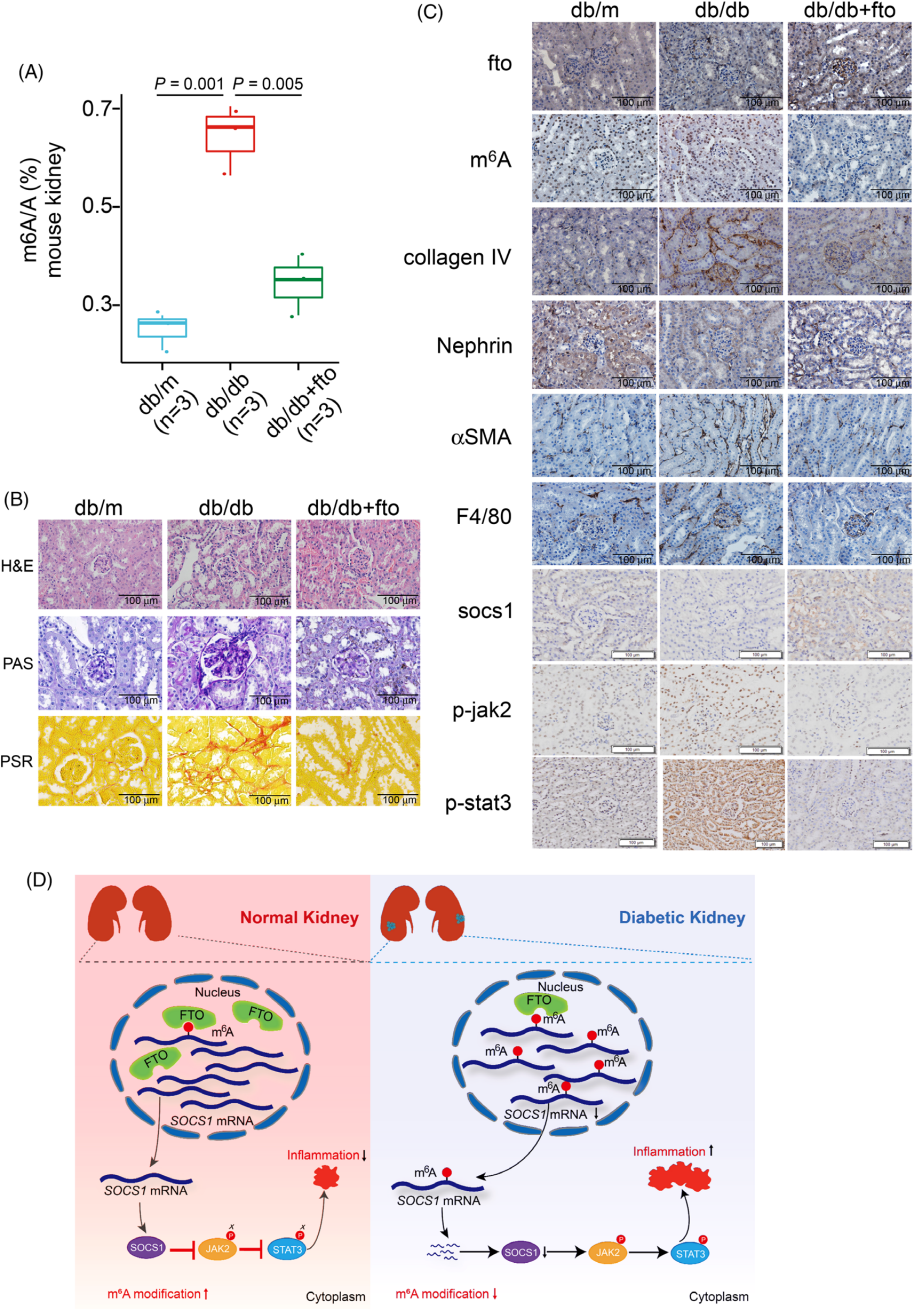
Fto overexpression alleviates kidney injury through increasing suppressors of cytokine signalling 1 (socs1) expression and decreased phosphorylation of jak2 and stat3 in vivo: (A) relative m^6^A levels in the kidney of *db*/*m*, *db*/*db* injected with control virus, and *db*/*db* with fto overexpression lentivirus; (B) representative images of H&E, PAS, and PSR analyses of kidneys from *db*/*m*, *db*/*db* injected with control virus, and *db*/*db* with fto overexpression lentivirus; (C) representative images of IHC analyses of indicated proteins of kidneys from *db*/*m*, *db*/*db* injected with control virus, and *db*/*db* with fto overexpression lentivirus; (D) model depicting the effect of decreased expression of m^6^A modulators in diabetic kidney disease (DKD): induction of a generalized state of hypermethylation and downregulation of socs1 and leading to increased phosphorylation of jak2 and stat3 and consequent kidney injury. Data are represented as mean  ± s.e.m. Statistical analyses were performed by two‐tailed unpaired student *t*‐tests and corrected for multiple comparisons using the Holm‐Sidak method.

In conclusion, our findings reveal a protective role of FTO during DKD pathogenesis. Mechanistically, the FTO/SOCS1/JAK‐STAT axis promotes DKD pathogenesis via promoting inflammation (Figure 4D). Moreover, FTO expression is significantly decreased in DKD, and overexpression of FTO can dramatically alleviate kidney inflammation. Therefore, we suggest that therapeutic targeting of FTO in combination with current therapeutic approaches might be a new avenue for DKD treatment.

## DISCLOSURES

No potential conflicts of interest relevant to this article were reported.

## Supporting information

Supporting Information
**Figure S1 Quality evaluation of IMC analysis of human kidney tissues: (a)** The dot bolt analysis of synthetic RNA fragments with or without m^6^A modification; **(b)** summary example images of all 10 markers from different patients of the analysed cohort; (**c)** *t*‐SNE plot showing the batch effect and the expression of Vimentin, aSMA, Nephrin, CD68, Aquaporin II, Collagen IV, and E‐cadherin in each type of cell.
**Figure S2 Detection of m^6^A level by using LC‐MS/MS method: (a)** The calibration curve of m^6^A (top panel) and A (bottom panel) detected by LC‐MS/MS; (**b)** the chromatograms of A (red) and m^6^A (blue) in serum samples of T2D, DKD, and healthy volunteers detected by LC‐MS/MS.
**Figure S3 Decreased FTO expression levels are correlated with increased m^6^A modification levels in DKD patients**: (**a)** overall m^6^A levels in serum samples of T2D, DKD, and healthy volunteers by LC–MS/MS; (**b)** heat map shows m^6^A regulators expression pattern in 15 serum samples. Five replicates for each group; (**c)** overall m^6^A levels in serum samples of T2D, DKD, and healthy volunteers from another cohort by LC–MS/MS; (**d)** qPCR analyses of FTO expression in serum samples of T2D, DKD, and healthy volunteers from cohort 2; (**e)** correlation between m^6^A levels and *FTO* mRNA expression in serum samples of T2D, DKD, and healthy volunteers from cohort 2; (**f)** the expression of *FTO* mRNA levels in blood samples of healthy volunteers and uraemia patients from GSE37171 dataset; (**g,h)** the expression of *FTO* mRNA levels in glomeruli of healthy volunteers and DN patients from GSE96804 **(g)** and GSE30122 **(h)** datasets. Data are represented as mean ± s.e.m. Statistical analyses were performed by two‐tailed unpaired student *t*‐tests and corrected for multiple comparisons using the Holm‐Sidak method.
**Figure S4 Expression levels of m^6^A modification regulators in T2D, DKD, and healthy volunteers**: (**a)** PCA plot of RNA sequencing after regressing out batch, sex and age in controls, T2D, and DN serums (*n* = 5 for each group); **(b and c)** qPCR analyses of YTHDC2 **(b)** and HNRNPA2B1 **(c)** expression in serum samples of T2D, DKD, and healthy volunteers from cohort 2; (**d)** qPCR analyses of key m^6^A modification regulators expression in serum samples of T2D, DKD, and healthy volunteers from cohort 1. Data are represented as mean ± s.e.m. Statistical analyses were performed by two‐tailed unpaired student *t*‐tests and corrected for multiple comparisons using the Holm‐Sidak method.
**Figure S5 High‐concentration glucose triggers m^6^A via reducing FTO expression**: (**a)** qPCR analyses of *FTO* mRNA expression in HMC or HK‐2 cells after treatment with high‐concentration glucose, normal‐concentration glucose, or mannitol; **(b)** Western blot analyses of indicated proteins in HMC or HK‐2 cells after treatment with high‐concentration glucose, normal‐concentration glucose, or mannitol; (**c)** overall m^6^A levels in HMC and HK‐2 cells after treatment with high‐concentration glucose, normal‐concentration glucose, or mannitol by LC–MS/MS; (**d)** correlation between m^6^A levels and FTO protein expression in HK‐2 and HMC cells; (**e)** Western blot analyses of indicated proteins in HMC or HK‐2 cells after FTO overexpression; (**f)** overall m^6^A levels in HMC and HK‐2 cells after FTO overexpression by LC–MS/MS; (**g)** Western blot analyses of indicated proteins in HMC or HK‐2 cells after FTO knock‐down; (**h)** overall m^6^A levels in HMC and HK‐2 cells after FTO knock‐down by LC‐MS/MS; (**i)** the ChREBP binding motif presented by the JASPAR database and schematic illustration of the potential binding sites of ChREBP on the promoter of FTO. Data are represented as mean ± s.e.m. Statistical analyses were performed by two‐tailed unpaired student *t*‐tests and corrected for multiple comparisons using the Holm‐Sidak method.
**Figure S6 Expression levels of m^6^A modification regulators in HMC and HK‐2 cells after HG treatment**: (**a–d)** qPCR analyses of *METTL3* **(a)**, *YTHDC2* **(b)** and *HNRNPA2B1* **(c)**, and *ALKBH5* **(d)** expression in HMC or HK‐2 cells after treatment with high‐concentration glucose, normal‐concentration glucose, or mannitol. Data are represented as mean ± s.e.m. Statistical analyses were performed by two‐tailed unpaired student *t*‐tests and corrected for multiple comparisons using the Holm‐Sidak method.
**Figure S7 SOCS1 is the potential target of FTO**: (**a)** m^6^A abundances on *SOCS1* mRNA transcripts in MONOMAC‐6 cells as detected by MeRIP‐seq from GSE76414 were plotted; (**b)** correlation between *SOCS1* and *FTO* mRNA expression in serum samples of T2D, DKD, and healthy volunteers from cohort 2; (**c)** Western blot analyses of indicated proteins in HMC or HK‐2 cells after FTO knock‐down; (**d)** Western blot analyses of indicated proteins in HMC or HK‐2 cells after treatment with high‐concentration glucose, normal‐concentration glucose, or mannitol; (**e)** Western blot analyses of indicated proteins in HMC cells after overexpressing empty vector, FTO wild‐type or mutant plasmid. Data are represented as mean ± s.e.m. Statistical analyses were performed by two‐tailed unpaired student *t*‐tests and corrected for multiple comparisons using the Holm‐Sidak method.
**Figure S8 Loss of SOCS1 promotes phosphorylation of JAK2 and STAT3**: (**a)** Protein–protein interaction network of SOCS1 according to STRING analysis; (**b)** GSEA results of significantly altered pathways after FTO overexpression base on RNASeq data, NES, normalized enrichment score; (**c)** Western blot analyses of indicated proteins in HMC or HK‐2 cells after FTO overexpression; (**d)** Western blot analyses of indicated proteins in HMC or HK‐2 cells after FTO knock‐down; (**e)** Western blot analyses of indicated proteins in HMC or HK‐2 cells after treatment with high‐concentration glucose, normal‐concentration glucose, or mannitol; (**f)** Western blot analyses of indicated proteins in HMC after indicated treatments.
**Figure S9 In vivo experiments indicated the therapeutic value of fto: (a–c)** Body weight **(a)**, kidney weight **(b)**, and kidney index **(c)** of *db*/*m*, *db*/*db* injected with control virus, and *db*/*db* with ftooverexpression lentivirus; (**d–g)** urinary protein **(d)**, blood creatinine **(e)**, blood glucose **(f)**, and blood urea nitrogen **(g)** of *db*/*m*, *db*/*db* injected with control virus, and *db*/*db* with fto overexpression lentivirus; (**h)** relative m^6^A levels in the blood of *db*/*m* and *db*/*db* mice; (**i–m)** relative ftoexpression of kidney or glomeruli in different diabetic mouse models. Data are represented as mean ± s.e.m. Statistical analyses were performed by two‐tailed unpaired student *t*‐tests and corrected for multiple comparisons using the Holm‐Sidak method.
**Figure S10 Quantification of m^6^A and indicated protein levels of *db*/*m*, *db*/*db* injected with control virus, and *db*/*db* with fto overexpression lentivirus: (a)** Quantification of indicated protein in Figure 4 analysed by IHC results of *db*/*m*, *db*/*db* injected with control virus, and *db*/*db* with fto overexpression lentivirus; (**b)** Western blot analyses of indicated proteins of *db*/*m*, *db*/*db* injected with control virus, and *db*/*db* with fto overexpression lentivirus. Data are represented as mean ± s.e.m. Statistical analyses were performed by two‐tailed unpaired student *t*‐tests and corrected for multiple comparisons using the Holm‐Sidak method.

Supporting Information

Supporting Information

Supporting Information

## References

[1] Wiener D , Schwartz S . The epitranscriptome beyond m(6)A. Nat Rev Genet. 2021;22:119–131. 33188361 10.1038/s41576-020-00295-8

[2] Ramalingam H , Kashyap S , Cobo‐Stark P , et al. A methionine‐Mettl3‐N(6)‐methyladenosine axis promotes polycystic kidney disease. Cell Metab. 2021;33:1234–1247.e1237. 33852874 10.1016/j.cmet.2021.03.024 PMC8172529

[3] Giesen C , Wang HA , Schapiro D , et al. Highly multiplexed imaging of tumor tissues with subcellular resolution by mass cytometry. Nat Methods. 2014;11:417–422. 24584193 10.1038/nmeth.2869

[4] Singh N , Avigan ZM , Kliegel JA , et al. Development of a 2‐dimensional atlas of the human kidney with imaging mass cytometry. JCI Insight. 2019;4:e129477. 10.1172/jci.insight.129477 PMC6629112 31217358

[5] Lu YT , Ma XL , Xu YH , et al. A fluorescent glucose transport assay for screening SGLT2 inhibitors in endogenous SGLT2‐expressing HK‐2 cells. Nat Prod Bioprospect. 2019;9:13–21. 30387082 10.1007/s13659-018-0188-4 PMC6328422

[6] Abdul‐Wahed A , Guilmeau S , Postic C . Sweet sixteenth for ChREBP: established roles and future goals. Cell Metab. 2017;26:324–341. 28768172 10.1016/j.cmet.2017.07.004

[7] Li Z , Weng H , Su R , et al. FTO plays an oncogenic role in acute myeloid leukemia as a N(6)‐methyladenosine RNA demethylase. Cancer Cell. 2017;31:127–141. 28017614 10.1016/j.ccell.2016.11.017 PMC5234852

[8] Kubo M , Hanada T , Yoshimura A . Suppressors of cytokine signaling and immunity. Nat Immunol. 2003;4:1169–1176. 14639467 10.1038/ni1012

[9] Liau NPD , Laktyushin A , Lucet IS , et al. The molecular basis of JAK/STAT inhibition by SOCS1. Nat Commun. 2018;9:1558. 29674694 10.1038/s41467-018-04013-1 PMC5908791

[10] Sullivan KA , Hayes JM , Wiggin TD , et al. Mouse models of diabetic neuropathy. Neurobiol Dis. 2007;28:276–285. 17804249 10.1016/j.nbd.2007.07.022 PMC3730836

