# Supplementary material for: FTO‐mediated m6A modification of SOCS1 mRNA promotes the progression of diabetic kidney disease
Source: Clin Transl Med. 2022 Jun 22;12(6):e942. doi: 10.1002/ctm2.942 (PMC9217105; doi:10.1002/ctm2.942)
Supplement: Supplementary file 1 — Supporting Information Figure S1 Quality evaluation of IMC analysis of human kidney tissues: (a) The dot bolt analysis of synthetic RNA fragments with or without m6A modification; (b) summary example images of all 10 markers from different patients of the analysed cohort; (c) t‐SNE plot showing the batch effect and the expression of Vimentin, aSMA, Nephrin, CD68, Aquaporin II, Collagen IV, and E‐cadherin in each type of cell. Figure S2 Detection of m6A level by using LC‐MS/MS method: (a) The calibration curve of m6A (top panel) and A (bottom panel) detected by LC‐MS/MS; (b) the chromatograms of A (red) and m6A (blue) in serum samples of T2D, DKD, and healthy volunteers detected by LC‐MS/MS. Figure S3 Decreased FTO expression levels are correlated with increased m6A modification levels in DKD patients: (a) overall m6A levels in serum samples of T2D, DKD, and healthy volunteers by LC–MS/MS; (b) heat map shows m6A regulators expression pattern in 15 serum samples. Five replicates for each group; (c) overall m6A levels in serum samples of T2D, DKD, and healthy volunteers from another cohort by LC–MS/MS; (d) qPCR analyses of FTO expression in serum samples of T2D, DKD, and healthy volunteers from cohort 2; (e) correlation between m6A levels and FTO mRNA expression in serum samples of T2D, DKD, and healthy volunteers from cohort 2; (f) the expression of FTO mRNA levels in blood samples of healthy volunteers and uraemia patients from GSE37171 dataset; (g,h) the expression of FTO mRNA levels in glomeruli of healthy volunteers and DN patients from GSE96804 (g) and GSE30122 (h) datasets. Data are represented as mean ± s.e.m. Statistical analyses were performed by two‐tailed unpaired student t‐tests and corrected for multiple comparisons using the Holm‐Sidak method. Figure S4 Expression levels of m6A modification regulators in T2D, DKD, and healthy volunteers: (a) PCA plot of RNA sequencing after regressing out batch, sex and age in controls, T2D, and DN serums (n = 5 for each [file CTM2-12-e942-s001.docx]

**Methods**

**Patient serum samples**All samples were obtained with informed consent under a protocol approved by the Review Board of Xuzhou Medical University and the Ethics Committee of Xuzhou Center Hospital. Serum samples from healthy volunteers were obtained with age range of 18-70 years old. Inclusion criteria were fasting blood-glucose ≤ 6.0 mmol/L, fasting insulin around 2.6~24.9 μU/mL, postprandial 2h blood sugar ≤ 7.78 mmol/L, and body mass index (BMI) around 18.5~23.9 kg/m^2^. Serum samples from healthy volunteers were randomly selected and mixed to prepare the blank serum. Serum samples from patients with T2D and DKD were collected using following criteria: 1) for patients with new-onset T2D, fasting blood-glucose ≥ 7.0 mmol/L and urinary albumin excretion rate (UAER) <20 μg/min; 2) for DKD patients, UAER >100 μg/min. Exclusion criteria for all volunteers and patients were the presence of primary hypertension, gastric resection, other kidney disease (nephrotic syndrome, nephritis, and renal insufficiency et al.), hepatic injury, malignant tumor, lupus erythematosus, other endocrine diseases, and taking nephrotoxic or nucleoside drugs.

**RNA extraction form serum samples** All the serum samples were obtained from centrifugation of fasting blood and stored at -80 ^o^C. 1200 μL prechilled methano/chloroform (2:1) was added into 200 μL serum. After vortex for 2 min and sonicating for 1 min, the mixture was placed at -20 °C for 10 min. Then, the mixture was centrifuged with 20,000×g at 4 °C for 10 min, and the supernatant was dried using cryogenic freeze-drying concentrator. Next, the residue was re-dissolved using 100 μL of initial mobile phase. After vortex for 2 min and sonicating for 1 min, the solution was centrifuged with 20,000×g at 4 °C for 10 min. Total RNAs were isolated using the High Pure FFPE RNA Micro Kit (Invitrogen, USA) by following the manufacturer’s instructions. The RNA was then reverse-transcribed to cDNA using High-Capacity cDNA Reverse Transcription Kit (Thermo Fisher Scientific, USA) according to the manufacturer’s instructions

**Patient kidney biopsies.** All samples were obtained with informed consent under a protocol approved by the Review Board of Xuzhou Medical University and the Ethics Committee of Xuzhou Center Hospital. A total of 10 patients with DKD were diagnosed on the basis of urinary albumin-to-creatinine ratio (ACR) and renal function (calculated using the MDRD equation). Patients with UACR >300 mg/g, eGFR <90 mL/min/1.73 m^2^ were enrolled in the study. Kidney tissues were collected through ultrasound-guided kidney biopsy after informed consent was obtained (Supplementary Table 3).

**Histological and IHC staining**Human kidney tissues from DKD patients and health people were collected in the form of paraffin sections. These tissues are slices obtained from clinical biopsy. All samples were obtained with informed consent under a protocol approved by the Review Board of Xuzhou Medical University and the Ethics Committee of Xuzhou Center Hospital. For mouse tissues, kidney tissues were immediately dissected after execution. Then tissues were fixed in 10% formaldehyde solution for two days. Next, they were embedded in paraffin blocks using a Leica Asp300S (Leica Microsystems, Denmark). Sections were cut and dried at 65 °C for 1 h, and then stored at 4 °C until use. Hematoxylin and eosin (H&E) staining was used to show the histological changes between each group. Sirius red staining was used to show the collagen abnormality. For mesangial expansion analyses, sections were stained with Periodic-Acid Schiff's reagent (PAS) (Merck, USA) and imaged using microscope (Olympus, Japan).

Immunohistochemical (IHC) staining was performed by using the DAB substrate kit according to the manufacturer’s instructions (Vector Laboratories, USA). The dilution of each antibody used in IHC analyses is listed in Supplementary Table 1. Images were processed with ImageJ by following the software instructions. The IDO/area was used to represent the expression level of each protein.

**Protein extraction and western blotting**Cell lysates were ruptured with RIPA lysis buffer (Beyotime Biotechnology, China) containing PMSF, EDTA, and a cocktail of protease and phosphatase inhibitors (Roche, Switzerland), which were then subjected to western analyses with the indicated antibodies and IRDye 800CW-conjugated secondary antibodies. The signals were detected using LI-COR Odyssey system (LI-COR Biosciences, USA). The antibodies used in western blotting are listed in Supplementary Table 2.

**Imaging mass cytometry (IMC) for human tissues**For Antibody conjugation, an antibody panel was designed to target epitopes specific for kidney tissues to distinguish epithelial, mesenchymal and different cell types of kidneys (Supplementary Table 1). The antibodies were labeled with Lanthanide isotope tags by the Maxpar labeling kit (Fluidigm, USA) according to the manufacturer’s instruction. Antibody concentration and specificity were evaluated by visual inspection of IMC images of a variety of control tissues, including normal kidney and DKD kidney biopsies.

For Tissue antibody labeling, tissue samples were formalin-fixed and paraffin-embedded at the Xuzhou Medical University. The antibody panel described previously was used to stain the tissue sections. Tissue slides were baked at 60 ℃ for 2 h in the slide oven. The heating block was turn on 96 ℃. Then the slides were dewaxed in xylene overnight and rehydrated in a descending graded series of ethanol (ethanol: deionized water 100:0, 90:10, 80:20, 70:30, 50:50, 0:100; 5 min each). Heat-induced antigen retrieval was conducted in Tris-EDTA buffer at pH 9 for 30 min in 95 ℃ water bath. The slides were then and blocked with 3% BSA in TBS for 1 h after immediately cooling. Slides were then incubated with metal isotope tagged antibodies overnight at 4 ℃. Following incubation, slides were washed twice with TBS and air dried before IMC measurements.

For Imaging mass cytometry, images were acquired using a Hyperion Imaging Mass System (Fluidigm). The 700*700 square area from each tissue slide was laser-ablated in a rastered pattern at 200 Hz, and the ablated tissue aerosol was transported to a CyTOF mass cytometer (Fluidigm) for quantification as the manufacturer’s instruction described.

**Image processing, single-cell signal quantification, and Cell clustering**Data were converted to TIFF image format and segmented into single cells using the analysis pipeline described by Ali et al (https://github.com/BodenmillerGroup/ ImcSegmentationPipeline). Briefly, individual cells were segmented using a combination of Ilastik v.1.1.9 and CellProfiler v.2.1.1. Ilastik was used to generate a probability map by classifying pixels based on a combination of antibody stains to identify membranes and nuclei. Probability maps were then segmented into single-cell

object masks using CellProfiler. Single-cell protein abundance estimates corresponded to the mean ion count of all pixels encompassed by a cell area. For cell cluster, single-cell expression data were normalized to ^80^Ar before analysis, and then the expression data matrix was normalized by batch effects with combat function from R sva v.3.40.0 package. The Phenograph algorithm was then used to cluster all the cells based on normalized protein distribution values, resulting 22 clusters. Then the origin Phenograph clusters were manually combined to 5 dominant cell types of human kidney.

**Analysis of m^6^A/A ratio using HPLC-MS/MS**LC experiment for modified nucleosides were performed on Agilent 1260 HPLC (Agilent, USA) with Waters XBridge Amide column (2.1×150 mm, 3.5 μm). The column temperature was set at 35 ^o^C. The mobile phase was water containing acetic acid (99.9/0.1, *v/v*) and ammonium acetate (10 mmol/L solvent A), and ACN (solvent B). A gradient of 0 min, 95% (B); 10 min, 95% (B); 17 min, 92% (B); 20 min, 92% (B); 25 min, 90% (B); 30 min, 60% (B) was used. The post time was 5 min. The flow rate of mobile phase was set at 0.3 mL min^-1^. The injection volume >2 μL.

Mass spectrometric experiments were performed on Agilent 6460 triple-Q mass spectrometer (Agilent, USA). Agilent Data Analysis software version 5.0 was used for the data processing. The detection was performed under positive electrospray ionization (ESI) mode with multiple reaction monitoring (MRM). Solutions were infused from the ESI source at 0.3mL min-1 with parameters: capillary 4000 V, drying gas 11 L min^-1^, drying gas temperature 350 ^o^C. Nitrogen was used as the nebulizing and drying gas. All MS conditions were optimized to achieve maximal detection sensitivity. [D3]5-mdC was used as the isotope internal standard.

**Animal experiments**The 8-week old male BKS-db/db and db/m mice were obtained from Nanjing Biomedical Research Institute of Xuzhou Medical University (License Number: SCXK 2018-008). Fto over-expression db/db mice were generated through injecting the tail vein with Fto-overexpression lentivirus at 12 weeks. After 4 weeks, mice of three groups were executed to collect blood, urine, and kidney tissues. Blood glucose levels were measured using an automated glucose monitor (Bayer, Germany). Serum creatinine was detected by using Creatinine assay kit (Jincheng Bioengineering Institute, China) according to the manufacturer’s instructions. Urea assay Kit purchased from Jincheng Bioengineering Institute (China) was used to quantify levels of urea nitrogen and urine protein of mice in each group. Te modifications of m^6^A was determined using HPLC-MS/MS. RNA and protein level were detected by RT-qPCR, western blot and immunohistochemistry using the indicated primers and antibodies.

**qRT-PCR analysis**Total RNAs were isolated with TRIzol^TM^ reagent (Invitrogen, USA). 200 ng RNA was reverse-transcribed into cDNA in a 10 μl reaction with High-Capacity cDNA Reverse Transcription Kit (Thermo Fisher Scientific, USA) according to the manufacturer’s instructions. RNA concentration was measured by ultraviolet absorbance at 260 nm. RT-qPCR was used to assess the relative abundance of mRNA. qPCR was performed by using ChamQ SYBR qPCR Master Mix (Vazyme, China) in a LightCycler^®^ 480 II system (Roche, Switzerland). Actin was used as the endogenous control. All reactions were run in triplicate. The primers for qRT-PCR are listed in Supplementary Table 3.

**m^6^A RNA-IP-qPCR**Total RNAs were isolated with TRIzol. Then, the polyA+ RNA was separated and enriched using the NEBNext Poly(A) mRNA Magnetic Isolation Module according to the manufacturer’s instruction (New England Biolabs, USA). 200 ng purified mRNA was used for each RNA-immunoprecipitation reaction. m^6^A RNA immunoprecipitation was then performed using Magna MeRIP m^6^A kit according to the manufacturer’s instructions (17-10499, Millipore Sigma, USA). m^6^A modification enrichments of *SOCS1* were evaluated using qPCR with primers covering the potential m^6^A region. The β-actin gene was used as an internal control when carrying out qPCR. The relative *SOCS1* mRNA expression was calculated by the value of Ct in m^6^A IP sample divide by the value of Ct in input sample (Ct_IP_ / Ct_input_). The MYC gene is used as a negative control. Primers used in m^6^A RNA-IP-qPCR are listed in Supplementary Table 2.

**RNA immunoprecipitation (RIP) qPCR assays** RNA immunoprecipitation (RIP) assays were performed by using Magna RIP Kit (Millipore, New Bedford, MA) according to the manufacturer’s instructions. Cells were lysed with appropriate amount of complete RIP Lysis Buffer. RNA-binding protein were immunoprecipitated using anti-FTO antibody (Santa Cruz, USA) and normal rabbit IgG. The co-precipitated RNAs were purified and dissolved in RNase-free water. Binding RNA targets were analyzed using qRT-PCR.

**Bioinformatic analysis of public resources**Transcriptomes of blood or glomeruli based on microarray were obtained from Gene Expression Omnibus (GEO) database. Transcriptomes of kidney or glomeruli based on microarray were also obtained from GEO database. For Affymetrix platform array, the raw CEL files were downloaded and normalized with the MAS5 algorithm using Custom Chip Definition Files that map to mouse official Gene Symbol  (Brainarray version 23, <http://brainarray.mbni.med.umich.edu/>), the gene expression levels then went through log2 transformation. For arrays from other platforms, the processed probe-level expression files were downloaded and reannotated to gene-level expression data. Notably, if multiple probes targeted a certain gene, we averaged expression values of all probes as expression value of that gene. Datasets used in this study could be found in Supplementary Table 4.

For pathway enrichment analysis. Pathway and gene ontology enrichment analysis were performed using R package (version 3.16.0) with default settings. Protein–protein functional networks were constructed using string database with default settings.

**Construction of the stable cell lines**To construct the FTO knockdown and control cell lines, lentivirus for shFTO (Forward: 5’-CCGGTCACCAAGGAGACTGCTATTTCTCGAGAAATAGCAGTCTCCTTGGTGATTTTTG-3’, Reverse: 5’-AATTCAAAAATCACCAAGGAGACTG CTATTTCTCGAGAAATAGCAGTCTCCTTGGTGA-3’) and controls were packaged with PLKO.1 vector with anti-puromycin plasmid. For FTO overexpressing system, human FTO cDNA (NM_001363905.1) or mouse Fto cDNA (NM_011936.2) were cloned into pCDH puro lentiviral vector. Briefly, and 1.5 μg construct for overexpression or knockdown of specific genes were co-transfected into HEK-293T cells in 60mm cell culture dish with Effectene Transfection Reagent (301427, QIAGEN, USA). The lentivirus particles were harvested at 48 and 72 hours after transfection, and then filtered and titrated with 8 mg/ml Polybrene (TR-1003, Sigma, USA). Finally, the concentrated lentivirus particles were directly added into the target cells and incubated at 37 °C for 24 to 48 hours. The infected cells were screened by puromycin according to the instructions.

**Cell culture and treatment**Human glomerular mesangial cell line (HMC, source: male) and proximal tubule epithelial cell line (HK-2, source: male) used in this study were purchased from Cell Bank of Chinese Academy of Science (Shanghai, China). Cells were initially cultured in low-dose glucose DMEM medium (Gibco, USA) supplemented with 10% fetal bovine serum (FBS) (Gibco, USA) at 37 ^o^C with 5% CO_2_.

Cell density was adjusted to around 1×10^5^ cells/mL with low glucose DMEM medium and mixed by gently blowing. Then, 2 mL cell suspension was added to each well. After culturing for 12 hours, cells were cultured with high glucose (36 mmol/L), low glucose (5.56 mmol/L), and mannitol (5.56 mmol/L glucose and 30.44 mmol/L mannitol) DMEM medium containing 1% FBS for 96 hours until cellular extracts were measured.

**m^6^A immunoprecipitation sequencing and data analyses**Total RNAs were purified using TRIzol. RNA quality and concentration were measured using Agilent 2100 Bioanalyzer (Agilent Technologies, USA). Fragmented mRNA was incubated with anti-m^6^A polyclonal antibody (ABE572, Millipore, USA) for 2 hours at 4 ℃. Immunoprecipitated mRNAs or inputs were used for library construction using NEBNext ultra RNA library prepare kit for Illumina (New England Biolabs, USA). The libraries were sequenced on Illumina Novaseq 6000 platform (Illumina, USA) with 150 bp paired-end reads. Adaptors and low-quality reads were filtered using fastp (version 0.20.0) (39). Clean reads were aligned to UCSC hg38 reference genome using HISAT2 (version 2.1.0) (40) with parameter -k 1. R package exomePeak (version 1.6.0) was used to call and identify differentially methylated m^6^A peaks based on the input and m^6^A-IP bam files (41). Peaks were annotated using R package ChIPseeker (version 1.22.1) (42). Sequence motifs enriched in m^6^A peak regions compared to control regions were identified using HOMER (Version 4.9.1) (43) where the random peaks of 200 bp on human transcriptome were used as background sequence.

The input library is essentially an mRNA sequencing library. The analyses were identical to RNASeq analyses. R package ClusterProfiler (version 3.16.0) was used to perform enrichment analysis of significantly dysregulated expressed genes or m^6^A peaks (37).

**RNA-seq and data analyses**Total RNAs were isolated with TRIzol from serum samples. RNA quality and concentration were measured by using an Agilent 2100 Bioanalyzer. Samples with enough high-quality RNA were used for library preparation using NEBNext Ultra RNA Library PrepKit for Illumina according to instructions. Paired-end libraries were sequenced on Illumina NovaSeq 6000 platform, and 150-bp paired-end reads were generated. After removal of adaptors and low-quality reads using fastp (version 0.20.0) (39), RNA-seq reads were aligned to human reference sequence (hg38) using HISAT2 (version 2.1.0) (40). Raw counts were calculated using featureCounts (version 2.0.0) based on the annotation obtained from GENCODE (44, 45). StringTie (version 2.1.0) was used to quantify gene expression levels in TPM (Transcripts Per Kilobase Million) units (46).

**Statistics and reproducibility**Experiments were repeated at least three times with consistent results. Data are presented as the Mean ± SEM or Mean ± SD. Differences between groups were determined by using two-tailed Mann-Whitney U test or two-tailed Student’s *t*-test. Pearson correlation coefficients were calculated to evaluate correlation. The results of western blotting are the representative images of at least three independent experiments. For boxplots, the center line represents the median, the box limits show the upper and lower quartiles, and the outliers are represented as individual data points.

**Study approval**All animal experiments were conducted in accordance with the Association for Assessment and Accreditation of Laboratory Animal Care. All protocols were approved by the Institutional Animal Care and Use Committee of Xuzhou Medical University. All human studies and protocols used were approved by Xuzhou Medical University Committee on Human Studies.
