# Supplementary figures and images for: FTO‐mediated m6A modification of SOCS1 mRNA promotes the progression of diabetic kidney disease
Source: Clin Transl Med. 2022 Jun 22;12(6):e942. doi: 10.1002/ctm2.942 (PMC9217105; doi:10.1002/ctm2.942)

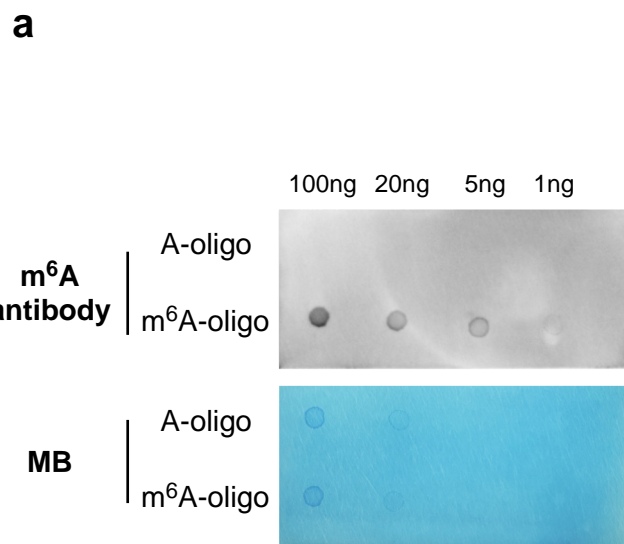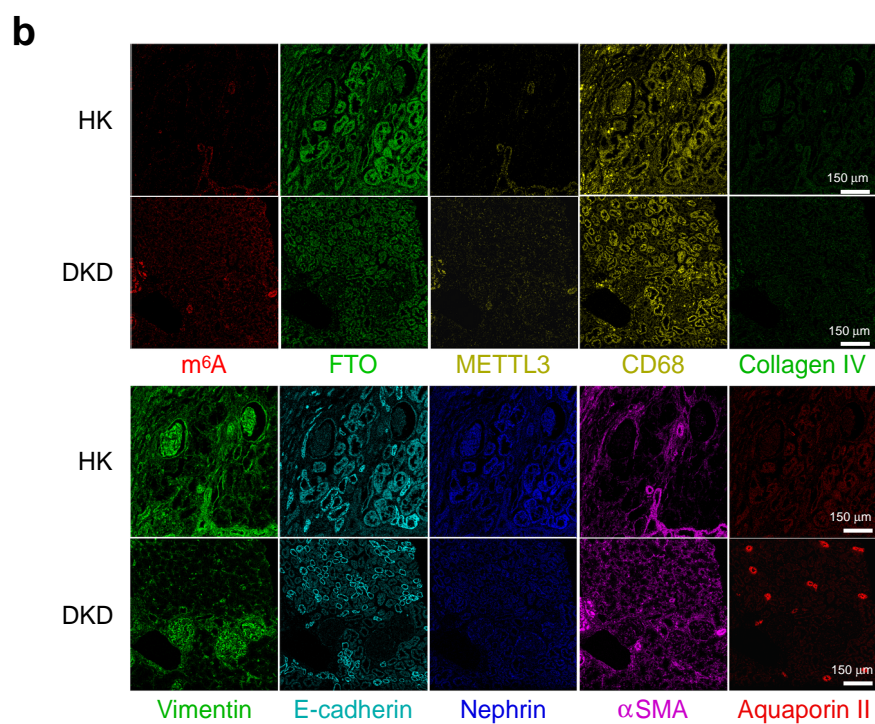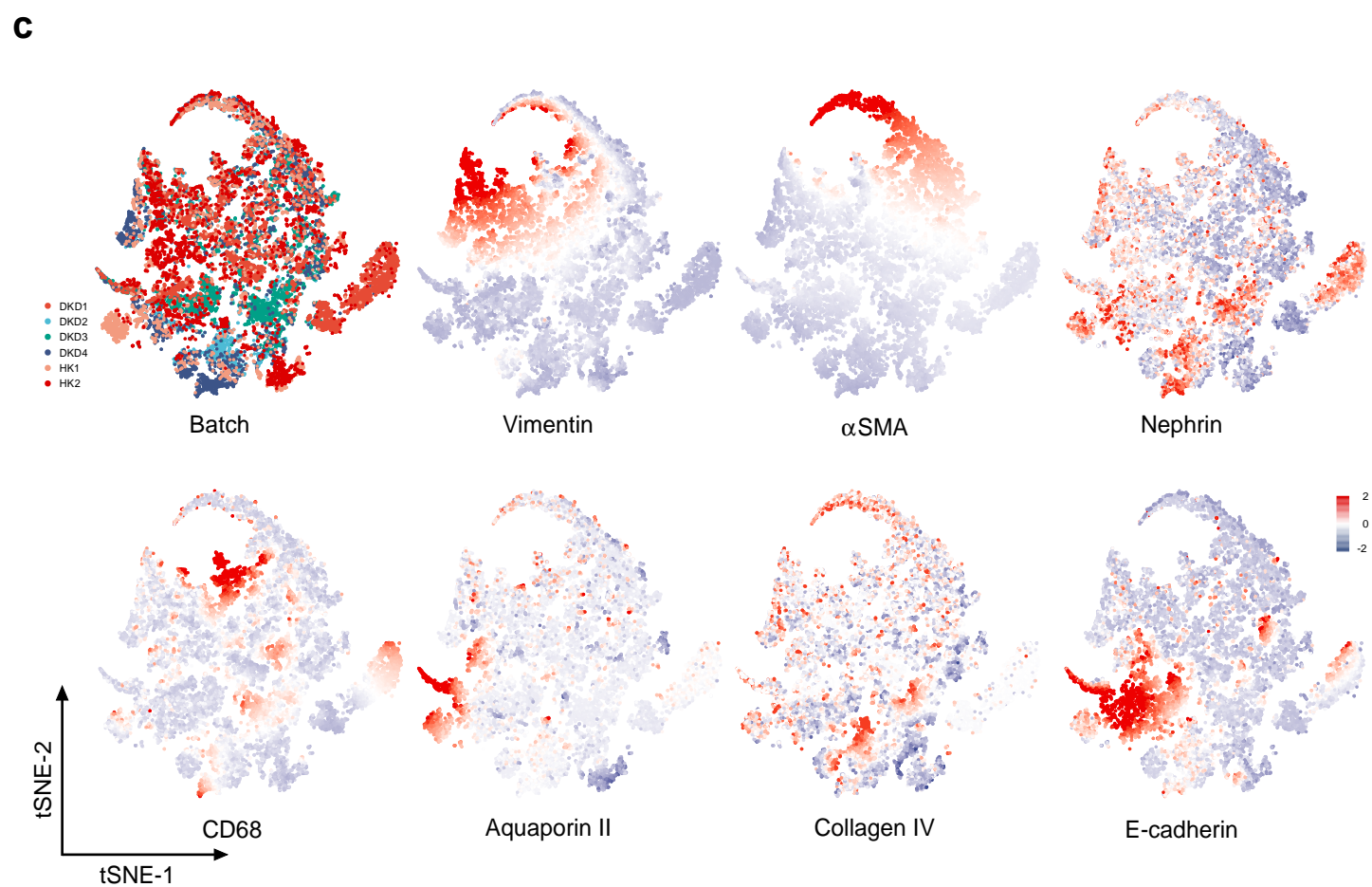

**a**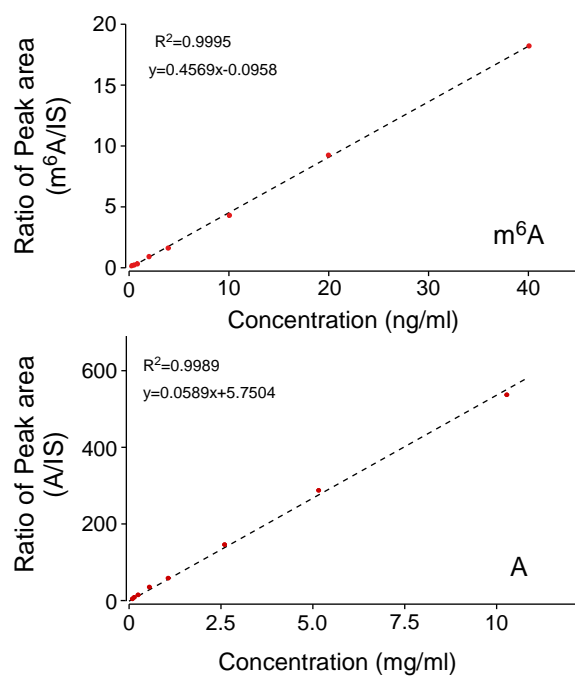**b**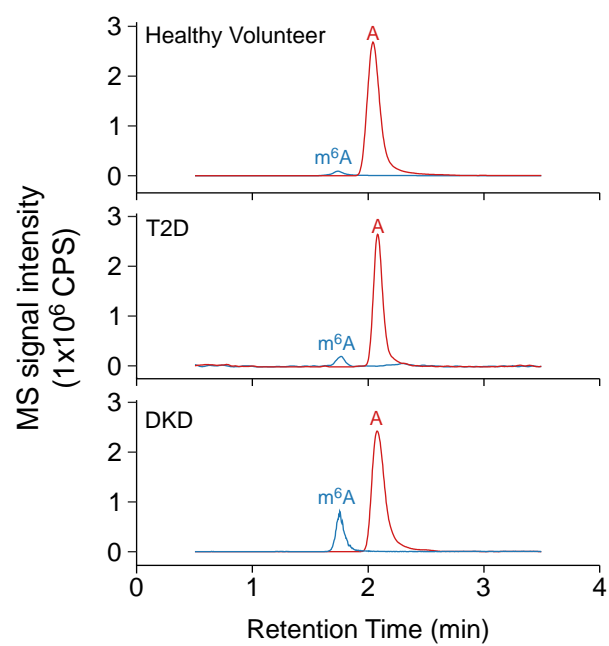

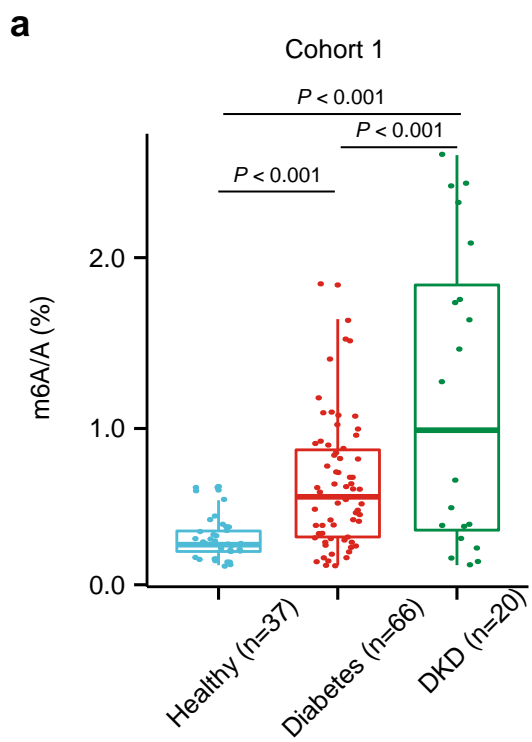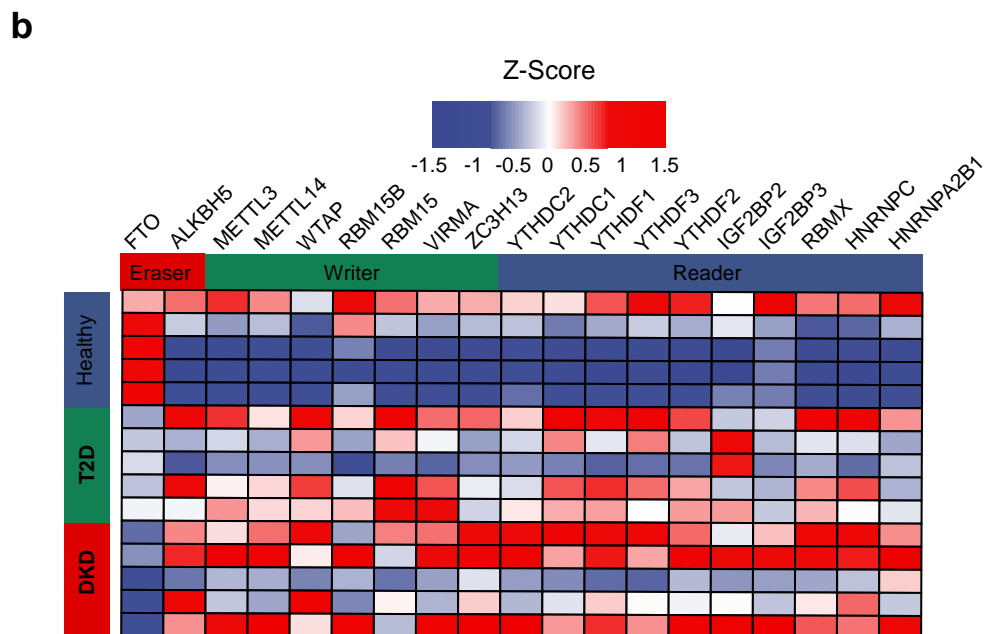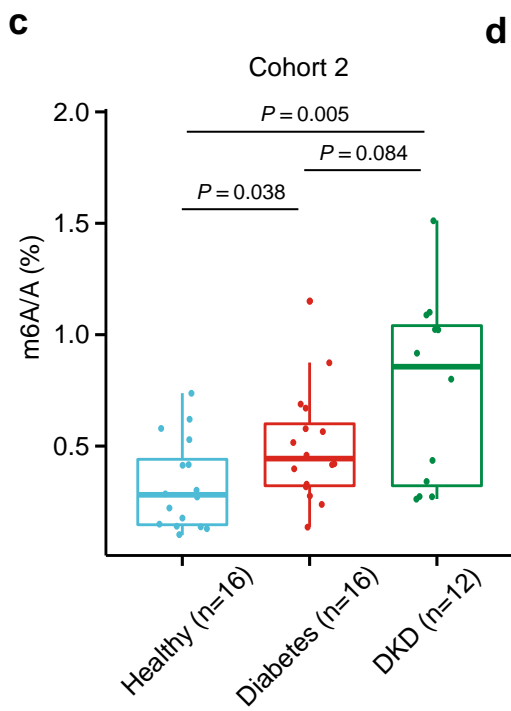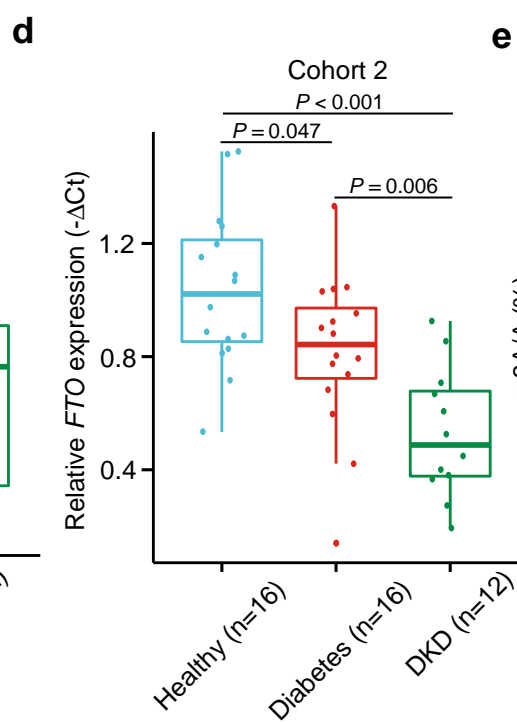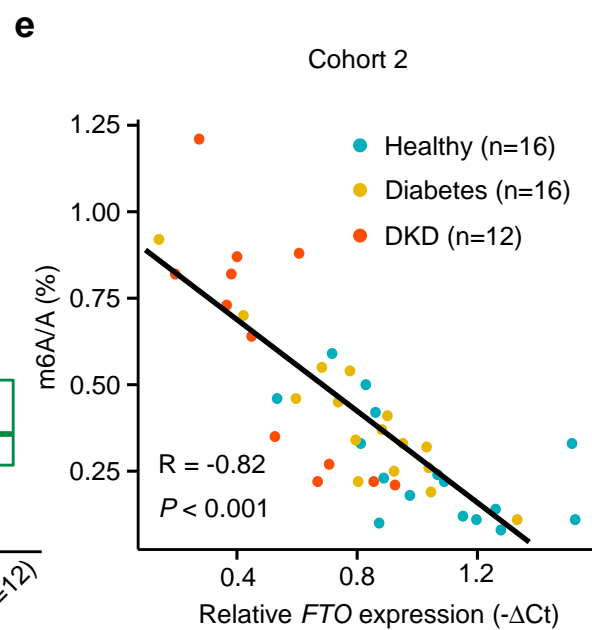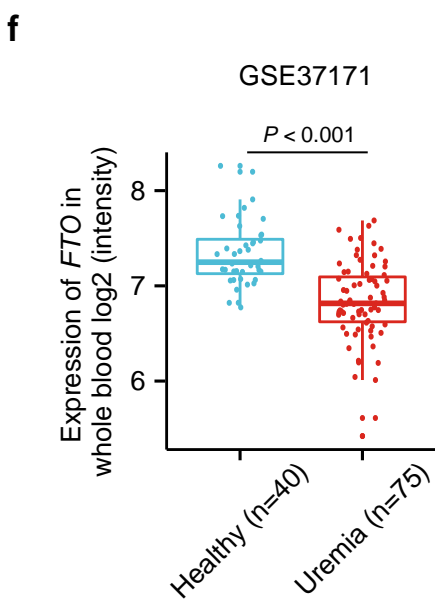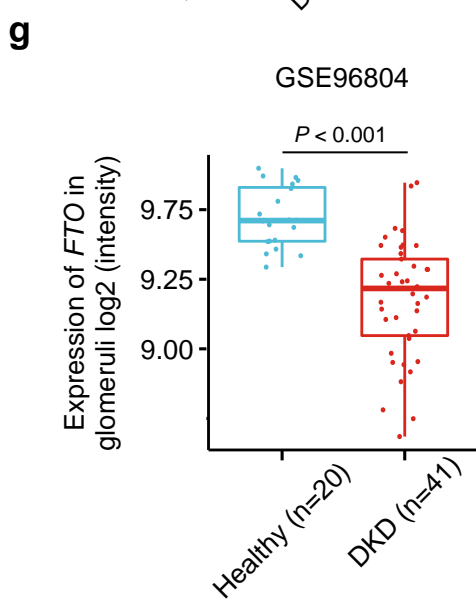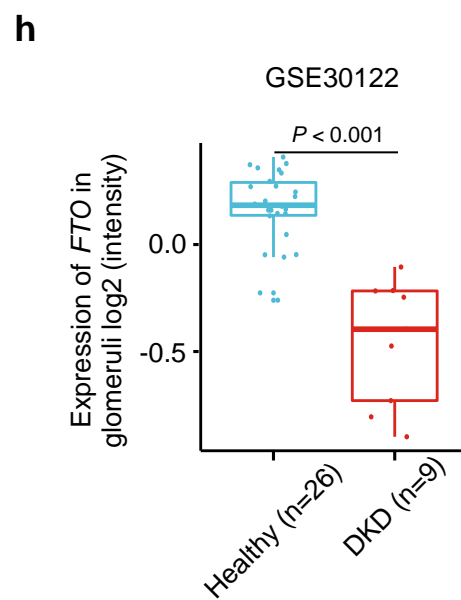

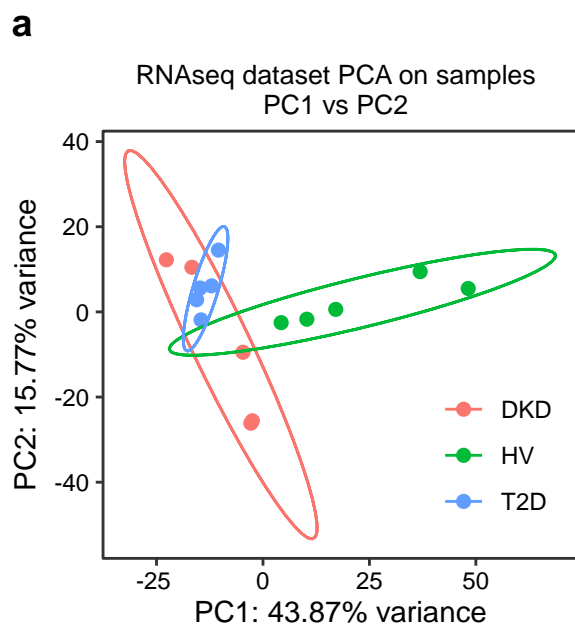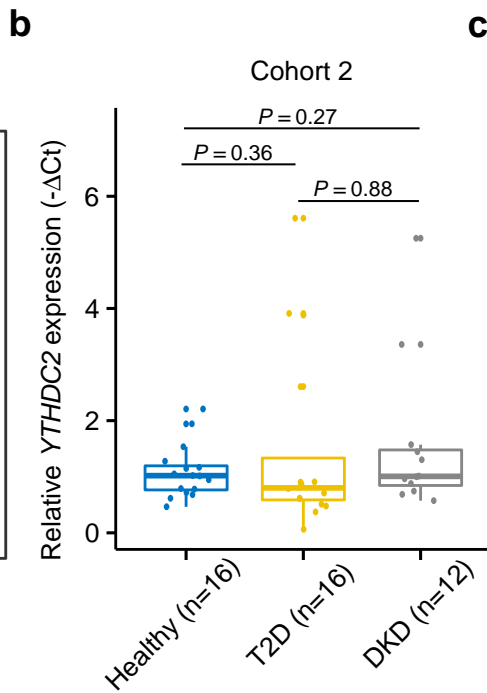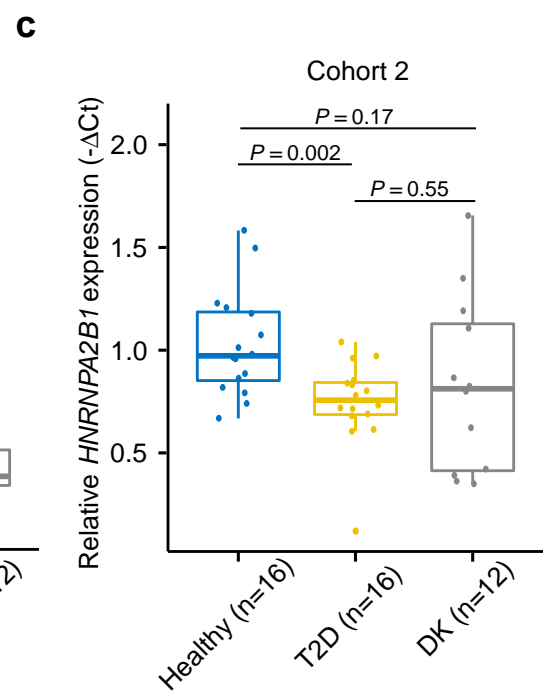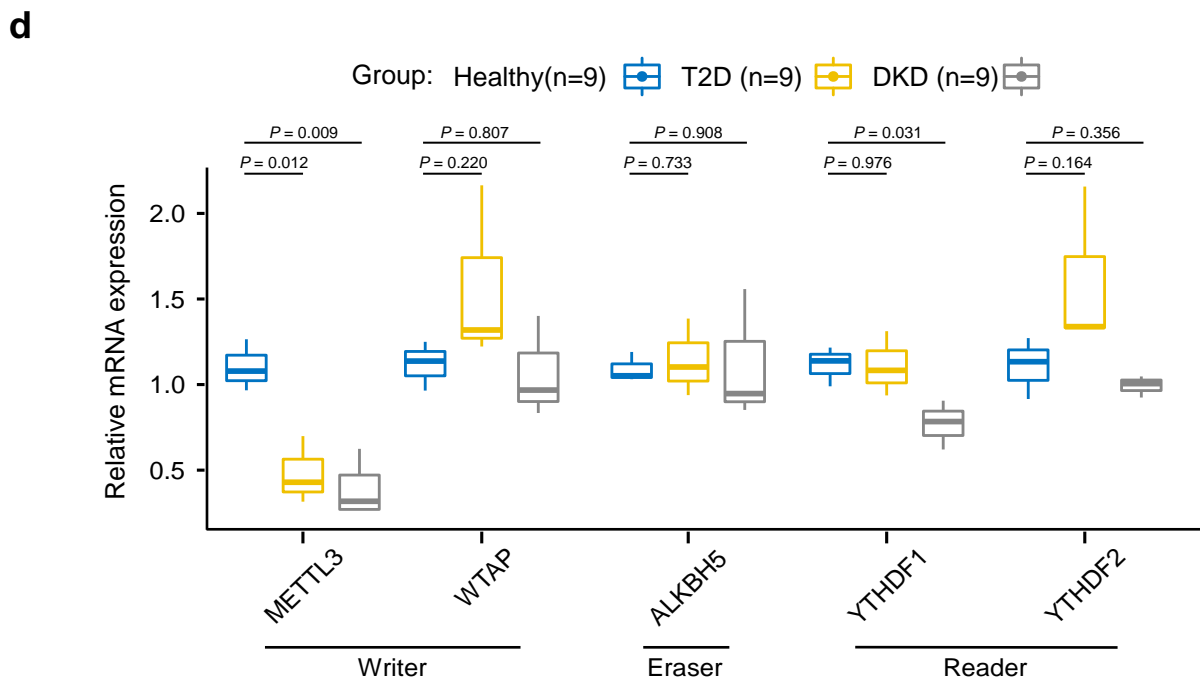

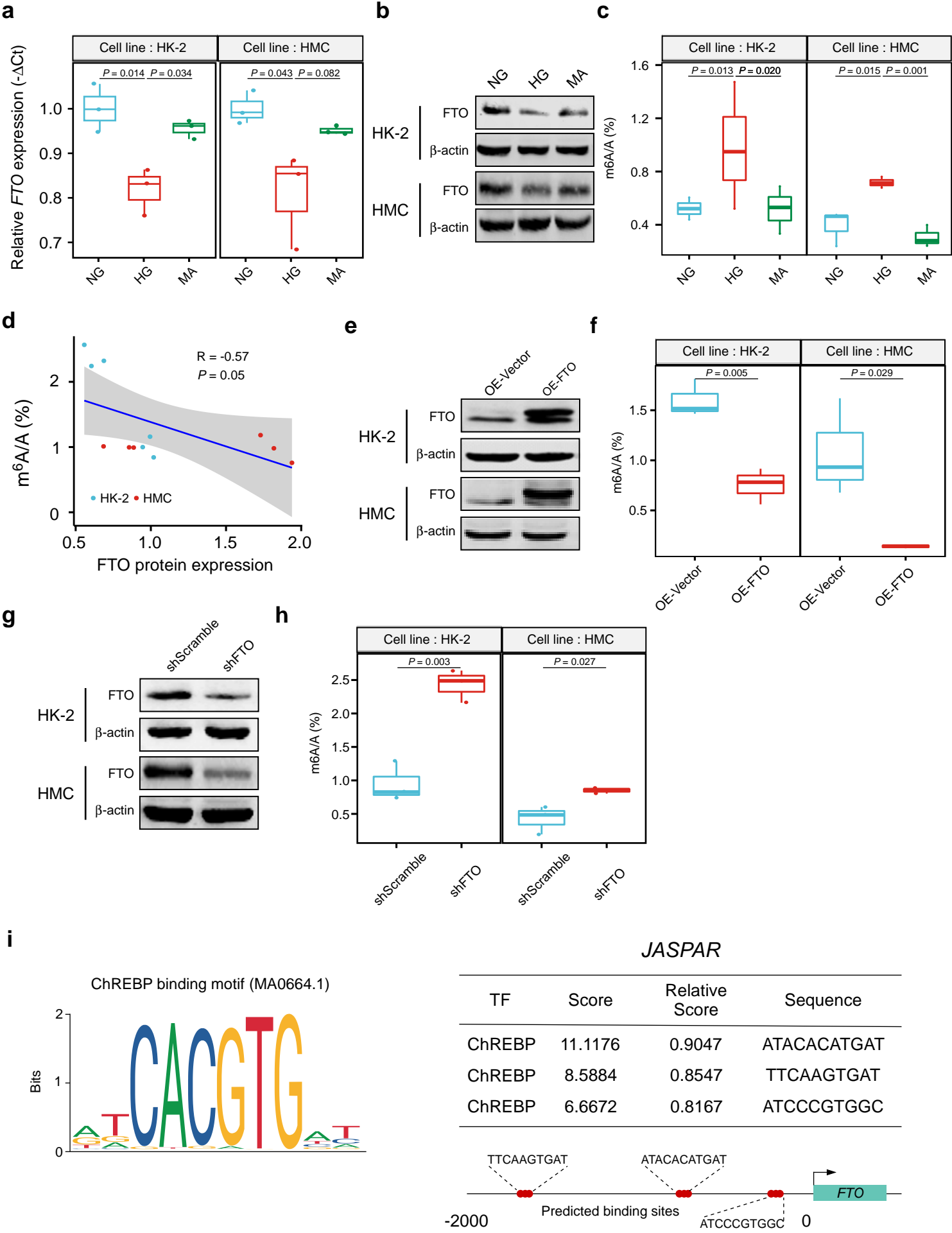

**a**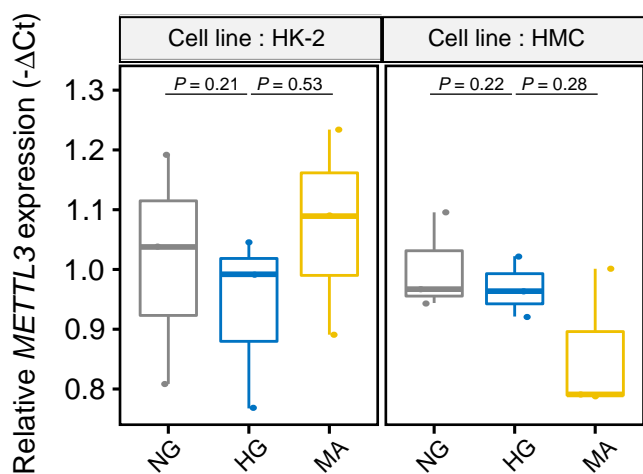**b**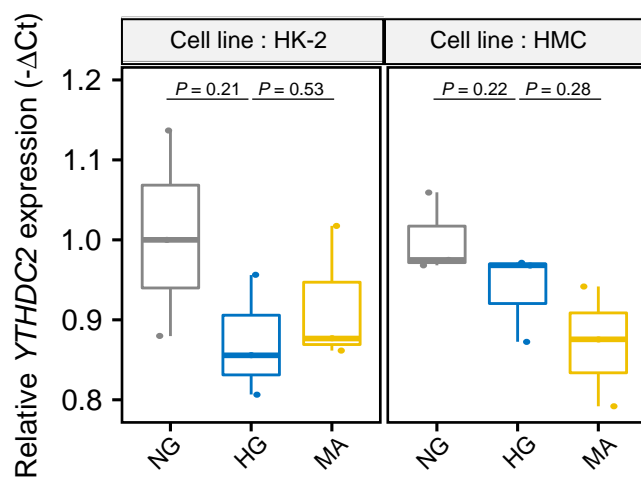**c**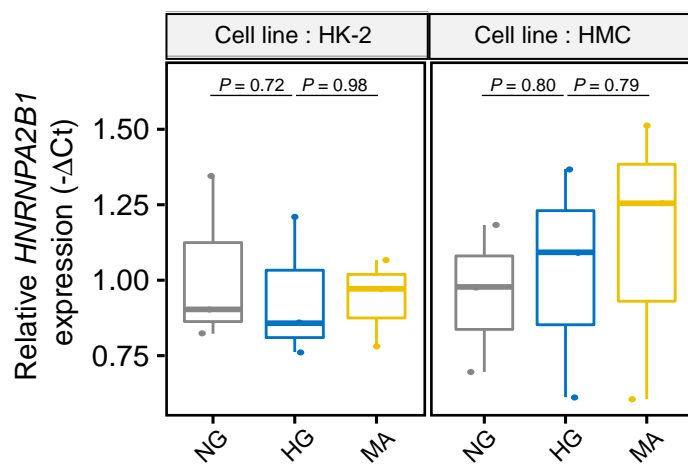**d**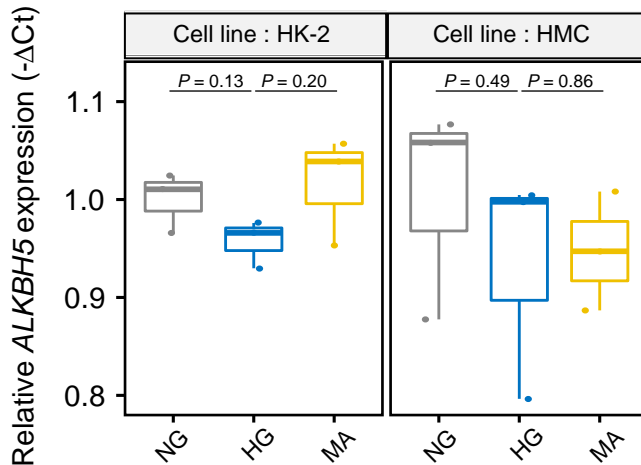

**a**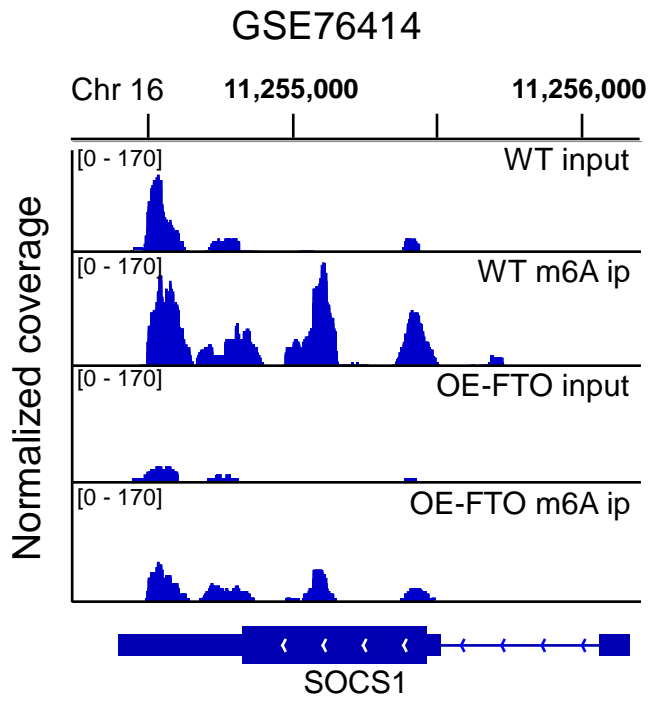**b**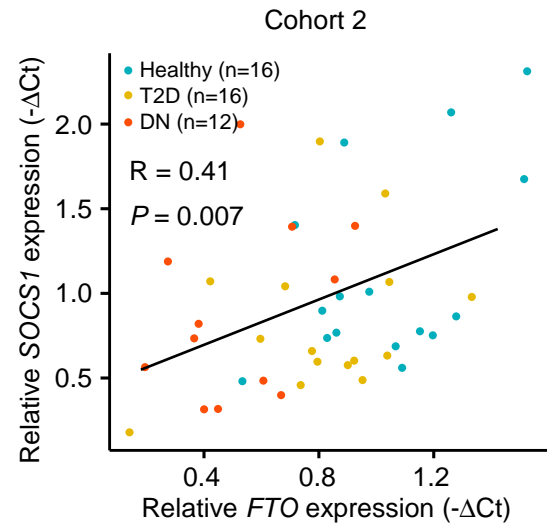**c**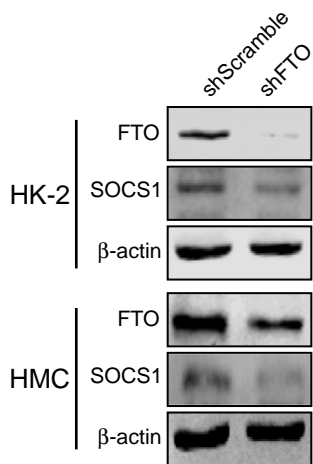**d**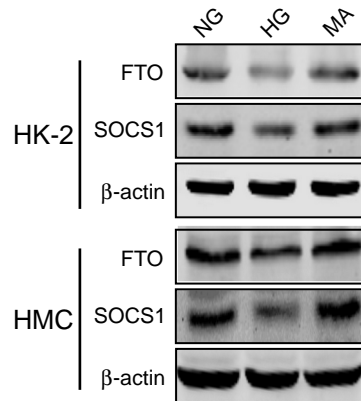**e**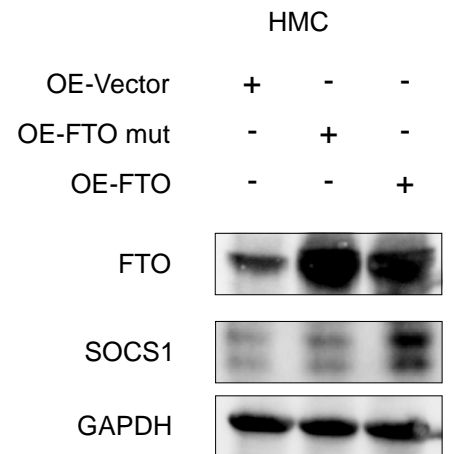

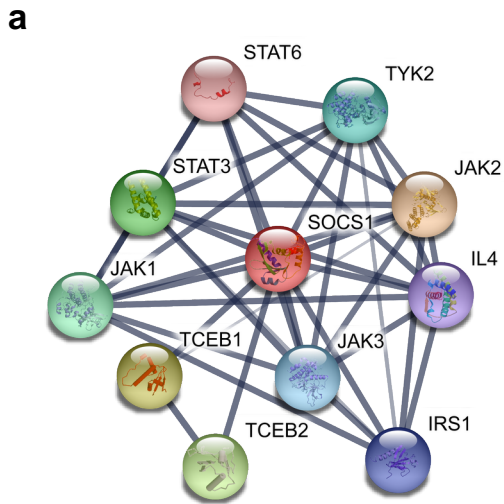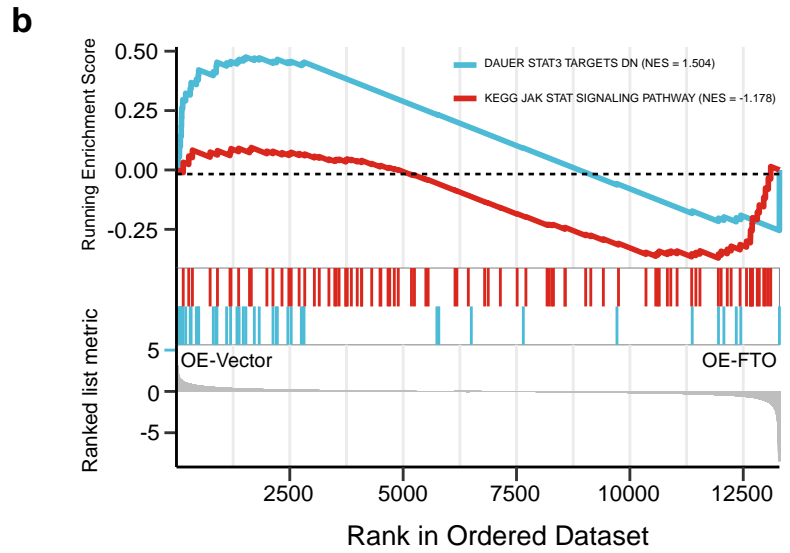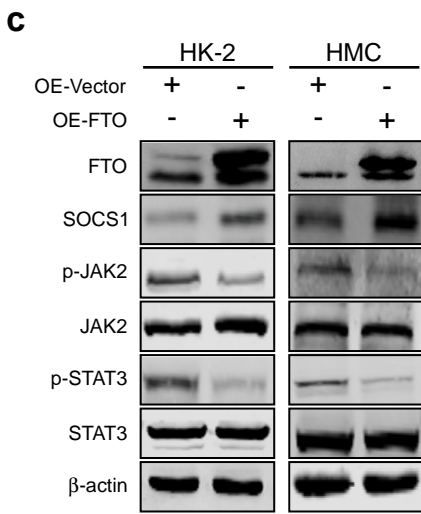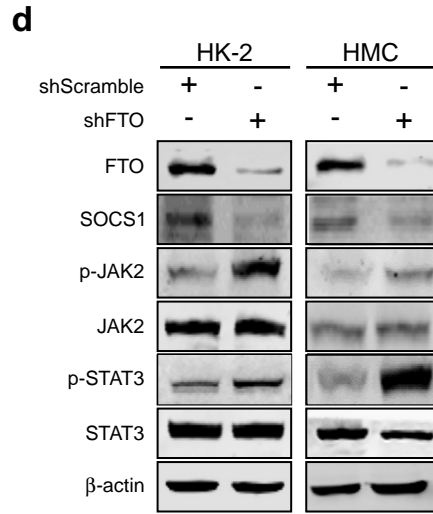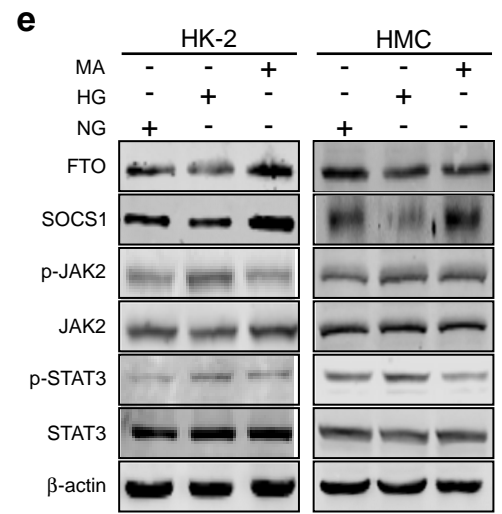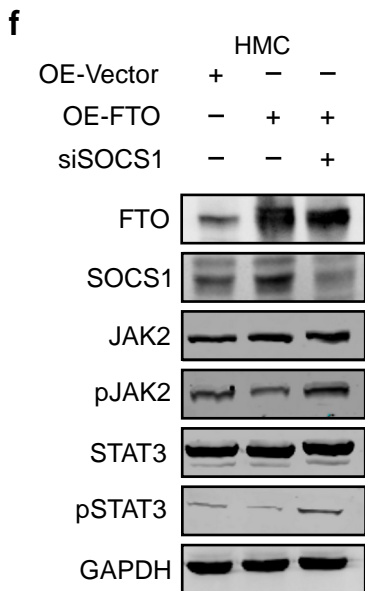

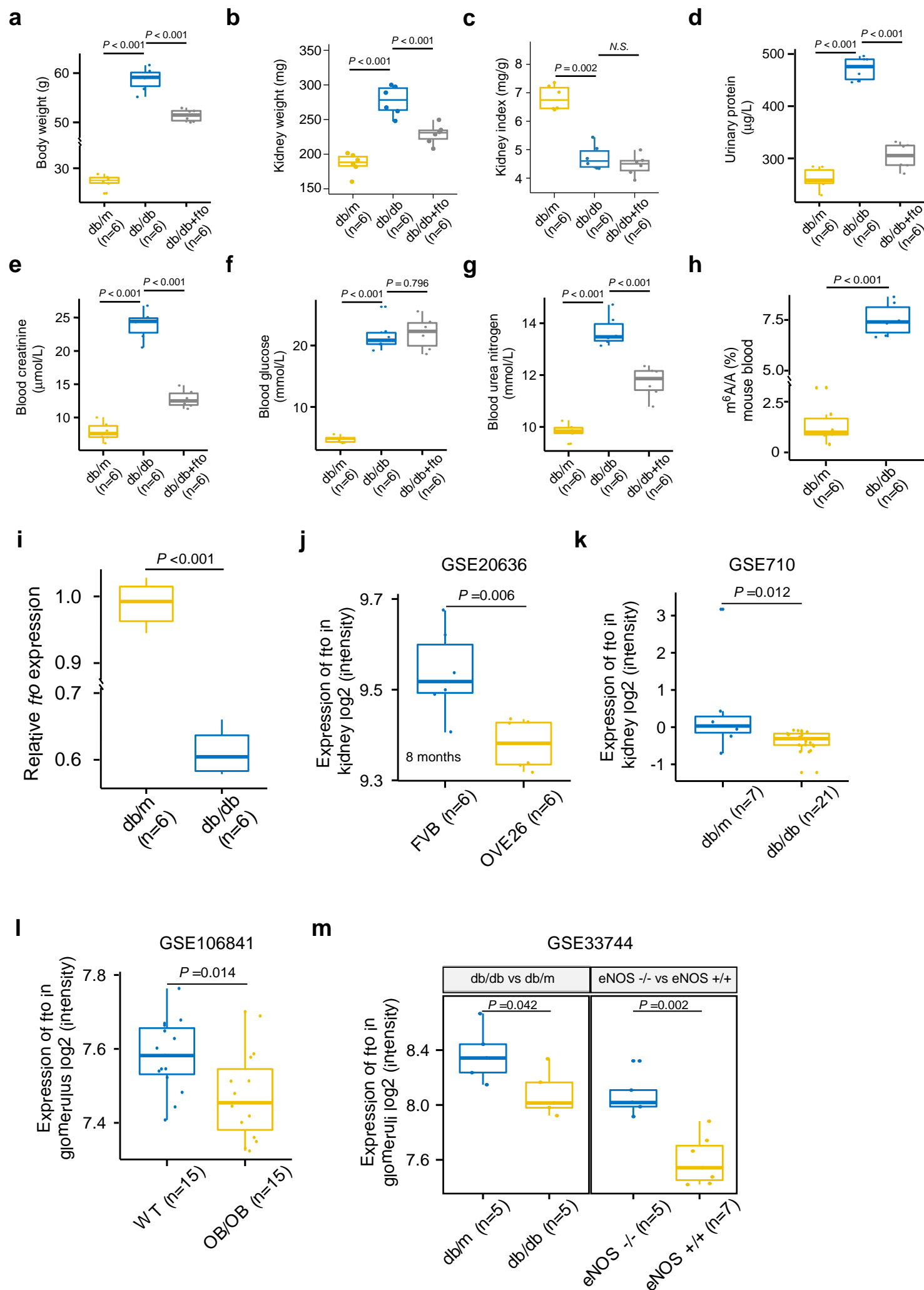

**a**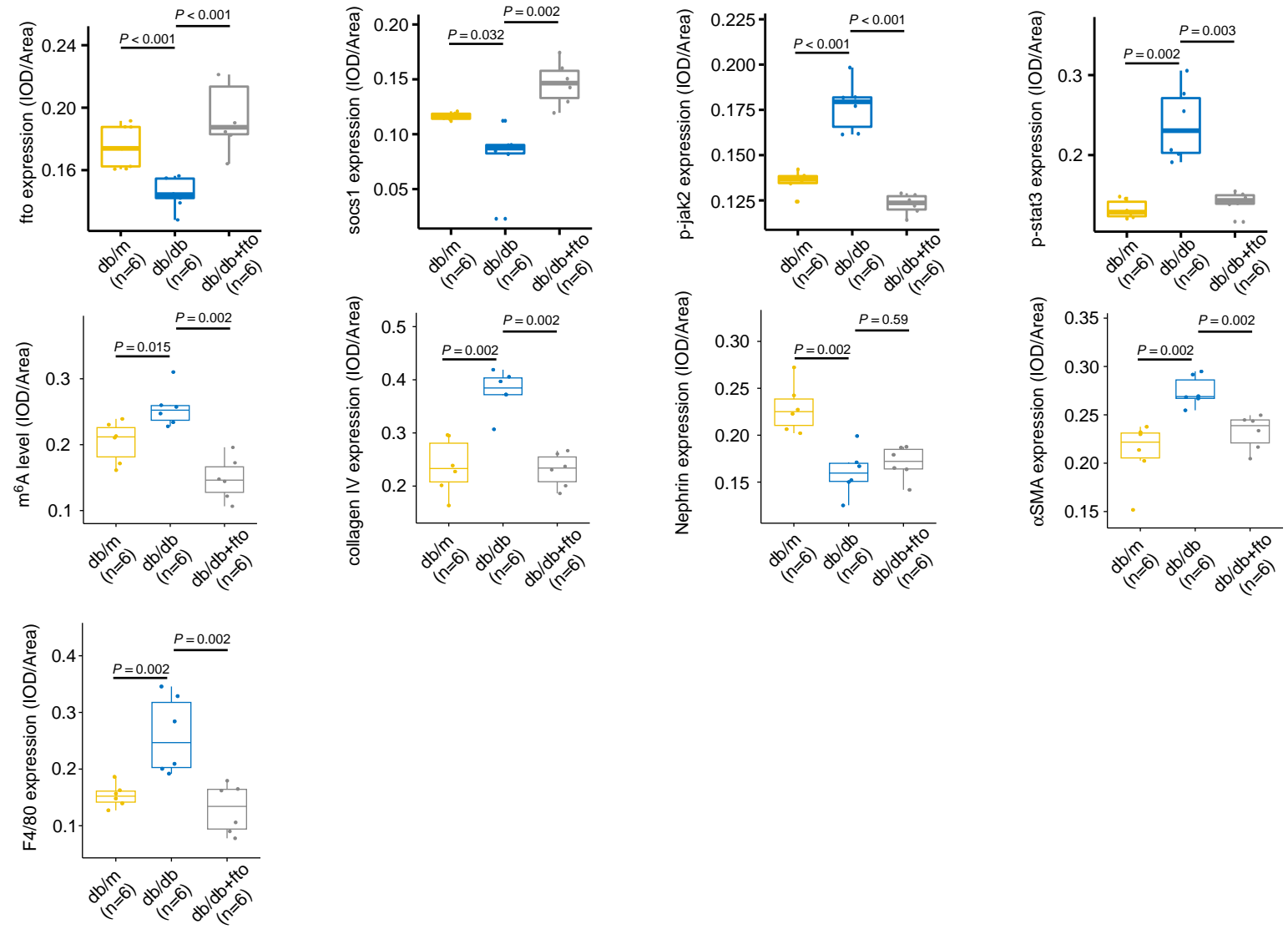**b**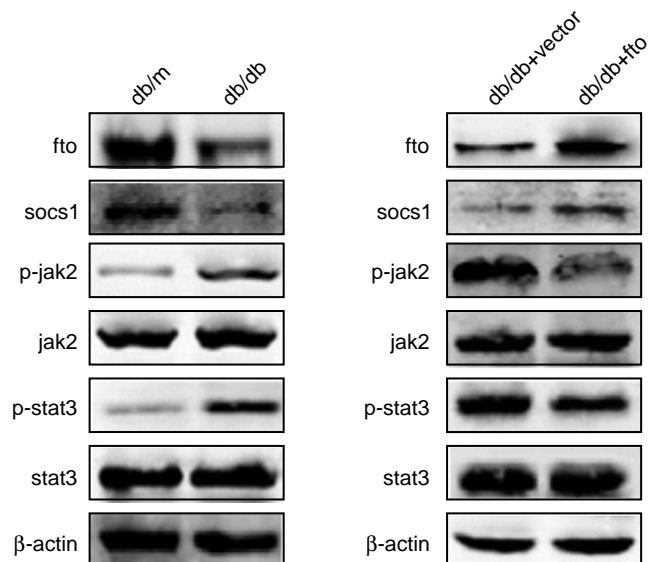

Supplement: Supplementary file 2 — Supporting Information [file CTM2-12-e942-s002.pdf]
