# Supplementary material for: FTO‐mediated m6A modification of SOCS1 mRNA promotes the progression of diabetic kidney disease
Source: Clin Transl Med. 2022 Jun 22;12(6):e942. doi: 10.1002/ctm2.942 (PMC9217105; doi:10.1002/ctm2.942)
Supplement: Supplementary file 3 — Supporting Information [file CTM2-12-e942-s004.docx]

**High glucose Increasing FTO-mediated m 6 A modification of promotes the progression of diabetic kidney disease**

**Supplementary Table 1 Antibody panel used in IMC experiments**

| Name | No. | Dilution | Manufacturer | Metal |
| --- | --- | --- | --- | --- |
| Anti-FTO antibody | ab92821 | 1:20 | Abcam | ^169^Tm |
| Anti-m^6^A antibody | 202003 | 1:20 | Synaptic | ^151^Eu |
| Anti-METTL3 antibody | ab221795 | 1:20 | Abcam | ^146^Nd |
| Anti-αSMA antibody | 1A4 | 1:100 | Fluidigm | ^141^Pr |
| Anti-Vimentin antibody | D21H3 | 1:100 | Fluidigm | ^143^Nd |
| Anti-Nephrin antibody | ab227806 | 1:20 | Abcam | ^149^Sm |
| Anti-E-cadherin antibody | 24E10 | 1:100 | Fluidigm | ^158^Gd |
| Anti-CD68 antibody | KP1 | 1:100 | Fluidigm | ^159^Tb |
| Anti-Aquaporin II antibody | ab230170 | 1:20 | Abcam | ^171^Yb |
| Anti-collagen IV antibody | ab256353 | 1:20 | Abcam | ^176^Yb |

**Supplementary Table 2 Antibodies used in western blotting and immunohistochemistry experiments**

| Name | No. | | Manufacturer | Application | Dilution |
| --- | --- | --- | --- | --- | --- |
| Anti-SOCS1 Antibody | 3950 | | CST | WB | 1:1000 |
| Anti-pSTAT3 (pY705) Antibody | ab76315 | | Abcam | WB; IHC | 1:2000; 1:50 |
| Anti-SOCS1 antibody | ab9870 | | Abcam | IHC | 1:100 |
| Anti-STAT3 antibody | ab68153 | | Abcam | WB | 1:1000 |
| Anti-FTO antibody | ab92821 | | Abcam | WB; IHC | 1:1000; 1:50 |
| Anti-JAK2 antibody | ab108596 | | Abcam | WB | 1:1000 |
| Anti-pJAK2 (pY1007/1008) antibody | 3776S | | CST | WB | 1:1000 |
| Anti-pJAK2 (pY1007/1008) antibody | ab32101 | | Abcam | IHC | 1:200 |
| Anti-Nephrin antibody | ab227806 | | Abcam | IHC | 1:1000 |
| Anti-Aquaporin II antibody | ab230170 | | Abcam | IHC | 1:100 |
| Anti-CD68 antibody | NB100-683 | | Novus Biologicals | IHC | 1:1000 |
| Anti-F4/80 antibody | 28463-1-AP | | Proteintech | IHC | 1:1000 |
| Anti-αSMA antibody | 14395-1-AP | | Proteintech | IHC | 1:1500 |
| Anti-β-actin Antibody | ac004 | | Abclonal | WB | 1:5000 |
| Anti-collagen IV antibody | ab256353 | | Abcam | IHC | 1:300 |
| Anti-m^6^A antibody | 202003 | | Synaptic | IHC | 1:100 |
| IRDye 800CW Goat anti-Rabbit IgG Secondary Antibody | V926-32211 | | VICMED | WB | 1:15000 |
| IRDye 800CW Goat anti-Mouse IgG Secondary Antibody | | V926-32210 | VICMED | WB | 1:15000 |

**Supplementary Table 3** Information of DKD patients with kidney biopsies

|  | ACR  (mg/g) | eGFR  (mL/min/1.73 m^2^) | Fasting blood glucose  (mmol/l) | Age  (y) | Gender |
| --- | --- | --- | --- | --- | --- |
| DKD01 | 455.9 | 47.20 | 12.23 | 56 | Male |
| DKD02 | 551.7 | 35.39 | 19.04 | 47 | Female |
| DKD03 | 398.1 | 53.95 | 9.86 | 62 | Female |
| DKD04 | 374.4 | 71.25 | 13.80 | 57 | Female |
| DKD05 | 308.5 | 87.92 | 8.01 | 61 | Male |
| DKD06 | 405.1 | 49.98 | 26.19 | 49 | Female |
| DKD07 | 379.5 | 76.73 | 9.16 | 51 | Male |
| DKD08 | 329.4 | 77.43 | 9.42 | 43 | Male |
| DKD09 | 303.6 | 88.99 | 8.55 | 39 | Female |
| DKD10 | 685.2 | 23.99 | 8.45 | 66 | Female |

**Supplementary Table 4 Primers used in this study**

| Gene | Species | Primer sequence (5’ to 3’) | Type |
| --- | --- | --- | --- |
| FTO | Homo  sapiens | FORWARD: CTTCACCAAGGAGACTGCTATTTC | qPCR |
|  |  | REVERSE: CAAGGTTCCTGTTGAGCACTCTG |  |
| ALKBH5 | Homo sapiens | FORWARD: AGGGGAAGCGTGACTGTGC | qPCR |
|  |  | REVERSE: GGGTGCATCTAATCTTGTCTTCC |  |
| METTL3 | Homo sapiens | FORWARD: ACGGAAGGTTGGAGACAATG | qPCR |
|  |  | REVERSE: GGACACGTGGAGCTCTATCC |  |
| METTL14 | Homo sapiens | FORWARD: ATTCCACTTGCCTGTGATGG | qPCR |
|  |  | REVERSE: CAGCAGGAATGTCACCTTGG |  |
| WTAP | Homo sapiens | FORWARD: TGGGAAGAGGTTCTTCGTTG | qPCR |
|  |  | REVERSE: TCCCTTCACCTTTCCTCTCC |  |
| YTHDF1 | Homo sapiens | FORWARD: TCGCTCATTGAGGGGTAACT | qPCR |
|  |  | REVERSE: GTGGACACCCAGAGAACAAAA |  |
| YTHDF2 | Homo sapiens | FORWARD: CTGCCTTTTATTTCCCACGA | qPCR |
|  |  | REVERSE: TGGAAAAGGCTAAGCAGGTG |  |
| YTHDC2 | Homo sapiens sapiens | FORWARD: CCACTTGTCTTGCTCATTTCCC | qPCR |
|  |  | REVERSE: CAAAACATGCTGTTAGGAGCCT |  |
| HNRNPA2B1 | Homo sapiens sapiens | FORWARD: AGCGACTGAGTCCGCGATGGA | qPCR |
|  |  | REVERSE: GCAGGATCCCTCATTACCACACAGT |  |
| β-Actin | Homo sapiens | FORWARD: CCTTGCACATGCCGGAG | qPCR |
|  |  | REVERSE: GCACAGAGCCTCGCCTT |  |
| SOCS1 | Homo sapiens sapiens | FORWARD: GCACGCAGCATTAACTGG | meRIP-PCR |
|  |  | REVERSE: TGTAAACATGAAGAGGTAGGAGG |  |
|  |  |  |  |
| MYC | Homo sapiens sapiens | FORWARD: TACAACACCCGAGCAAGGAC | meRIP-PCR |
|  |  | REVERSE: GCCTTTCAGAGAAGCGGGTC |  |
| β-Actin | Homo sapiens sapiens | FORWARD: ATGATATCGCCGCGCTCGT | meRIP-PCR |
|  |  | REVERSE: GGCGCCCCACGATGGA |  |

**Supplementary Table 5 Datasets included in this study**

| Dataset ID | No. Samples  (Healthy+Disease) | Platform | PMID | Type | Species |
| --- | --- | --- | --- | --- | --- |
| GSE37171 | 40+75 | Affymetrix HGU133 Plus 2.0 Array | 23809614 | Whole Blood Uremia | Homo sapiens |
| GSE96804 | 20+41 | Affymetrix Human Transcriptome Array 2.0 | 29242313 | Glomeruli DKD | Homo sapiens |
| GSE30122 | 26+9 | Affymetrix U133A 2.0 Array | 21752957 | Glomeruli DKD | Homo sapiens |
| GSE710 | 7+21 | cDNA spotted microarray | 14988265 | db/db | Mus musculus |
| GSE20636 | 6+6 | Affymetrix Mouse Genome 430 2.0 Array | 21606656 | OVE26 | Mus musculus |
| GSE106841 | 15+15 | Affymetrix Mouse Transcriptome Array 1.0 | 29340699 | OB/OB | Mus musculus |
| GSE33744 | 5+5/5+7 | Affymetrix Mouse Genome 430 2.0 Array | 23139354 | db/db  eNOS | Mus musculus |
